# Digital Cognitive Behavioral Therapy for Cardiac Anxiety After Myocardial Infarction: Effects on Disease-Specific Health Status–Detailed Methods and Protocol

**DOI:** 10.1016/j.jacadv.2026.102669

**Published:** 2026-03-25

**Authors:** Amanda Johnsson, Brjánn Ljótsson, Björn E. Liliequist, Matthias Lidin, Marie Löf, Linnea Maurex, Eva Ólafsdóttir, Elina Rautio, Johanna Sandborg, Frieder Braunschweig, Linda G. Mellbin, Josefin Särnholm

**Affiliations:** a Division of Psychology, Department of Clinical Neuroscience, Karolinska Institutet, Stockholm, Sweden; b Department of Medicine Solna, Karolinska Institutet, Stockholm, Sweden; c Department of Medicine Huddinge, Karolinska Institutet, Huddinge, Sweden; d Department of Cardiology, Heart and Vascular Center, Karolinska University Hospital, Stockholm, Sweden; e Center for Behavioral Cardiovascular Health, Columbia University Irving Medical Center, New York, New York, USA

**Keywords:** cardiac anxiety, digital cognitive behavior therapy, myocardial infarction, quality of life

## Abstract

**Background:**

Cardiac anxiety, characterized by cardiac-related fear and avoidance, is common after myocardial infarction (MI) and is associated with reduced quality of life and adverse prognosis. Despite its clinical relevance, cardiac anxiety remains underaddressed in routine post-MI care.

**Objectives:**

The objective of the study was to evaluate the efficacy of a therapist-guided, digitally delivered exposure-based cognitive behavioral therapy intervention for cardiac anxiety (CA-CBT) in patients after MI.

**Methods:**

In this randomized clinical trial, patients with a history of type 1 MI (≥6 months prior) and clinically significant cardiac anxiety were randomly assigned to 8 weeks of digital CA-CBT or usual care. The primary outcome was disease-specific health status assessed at 3-month follow-up using the Seattle Angina Questionnaire; cardiac anxiety was also assessed.

**Results:**

Compared with usual care, CA-CBT was associated with greater improvements in disease-specific health status, particularly quality of life and physical limitation, as well as greater reductions in cardiac anxiety. Treatment effects were sustained at 12 months.

**Conclusions:**

A therapist-guided, digitally delivered CBT intervention targeting cardiac anxiety improved disease-specific health status and reduced cardiac anxiety after MI, with durable effects. These findings support the clinical relevance of integrating psychological interventions into multidisciplinary secondary prevention after MI. (Online Cognitive Behavioral Therapy Targeting Cardiac Anxiety [MI-CBT]; NCT05580718)

## Graphical abstract

**Figure:**
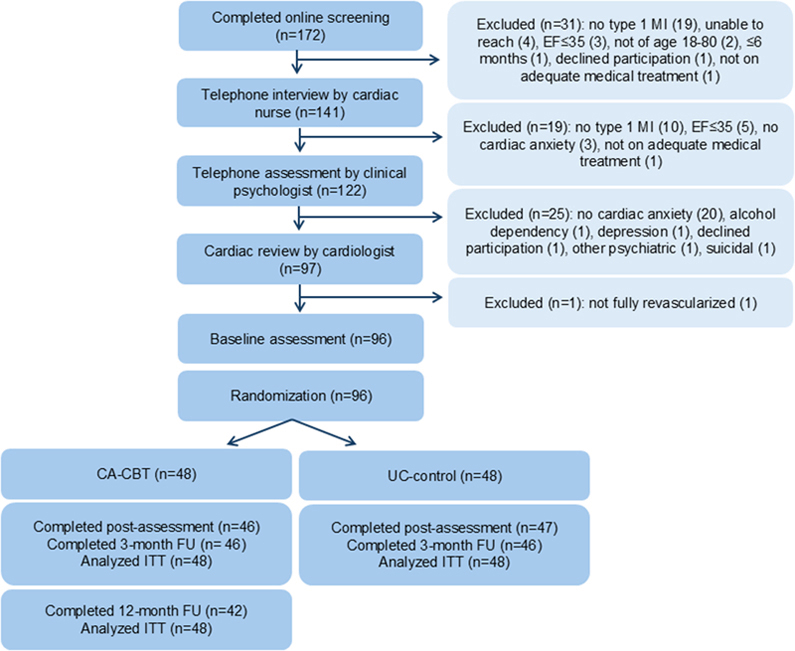

This paper describes the methods, trial design, and statistical analyses for a parallel-group randomized controlled trial (RCT) evaluating digital exposure-based cognitive behavioral therapy (CBT) for cardiac anxiety after myocardial infarction (MI). The main clinical results are presented in a companion Brief Report in *JACC*,^1^ whereas secondary outcomes are detailed and reported in this article.

MI remains a leading cause of health loss worldwide.^2^ Although survival has improved, years lived with disability have increased.^3^ Psychological distress is frequently observed after MI, with approximately 20 to 30% of patients reporting anxiety or depressive symptoms.^4^^,^^5^ This distress is clinically meaningful and associated with impaired recovery^5^ and adverse cardiovascular outcomes.^6^

Cardiac anxiety represents a disease-specific form of psychological distress after MI, characterized by excessive fear of recurrent cardiac events, hypervigilance to cardiac-related sensations (eg, chest discomfort, palpitations, or shortness of breath), and avoidance behaviors.^7^ Although precise prevalence estimates remain limited, available studies suggest that approximately 25% to 30% of post-MI patients experience clinically significant cardiac anxiety, with symptoms persisting over the first year in MI cohorts.^2^ Similar proportions have also been observed in broader coronary heart disease samples.^3^

Cardiac anxiety has been associated with lower engagement in health behaviors, adherence to secondary prevention measures,^8^^,^^9^ depressive symptoms,^8^ poor quality of life (QoL),^9^ and an adverse cardiac prognosis.^10^ After MI, cardiac-related sensations may become conditioned threat cues through interoceptive fear learning.^11^ Once associated with perceived threat, hypervigilance and avoidance of activities further reinforce threat beliefs and maintain heightened vigilance toward cardiac-related sensations,^7^ thus increasing the likelihood of misinterpretation of benign bodily fluctuations.^11^ Avoidance behavior may be overt (eg, reduced exercise and social participation) or covert (eg, self-monitoring or cognitive distraction).^12^ Although these behaviors may provide short-term relief, they maintain cardiac-related fear and contribute to persistent functional impairment over time.^7^^,^^10^^,^^13^

Psychological health is recognized as a modifiable risk factor and an essential component of comprehensive cardiovascular care.^14^^,^^15^ However, psychological interventions remain rarely integrated into secondary prevention.^16^ From a patient-centered perspective, this reflects a gap in care delivery and a missed opportunity to address psychological processes that influence recovery after MI. Existing psychological interventions in cardiac populations have largely focused on general anxiety and depression,^17^ potentially leaving cardiac-specific fear and avoidance behaviors insufficiently addressed. Exposure-based CBT is designed to interrupt fear-based patterns by promoting gradual engagement with feared cardiac-related sensations and previously avoided activities, that help the patient to re-engage in daily activities and improve QoL.^18^^,^^19^ Although exposure-based CBT is well established for anxiety disorders^20^ and effective in reducing fear and avoidance in other somatic conditions,^21^^,^^22^ it has been infrequently applied in cardiac population, partly due to concerns about safety in medically vulnerable patients^23^ as well as limited awareness of psychological mechanisms among cardiologists and insufficient integration between cardiology and psychological care services.^15^ Digitally delivered CBT offer a scalable approach that may facilitate broader implementation.^24^^,^^25^

Building on this rationale, Särnholm et al.^26^, ^27^, ^28^ developed and evaluated digital exposure-based CBT protocols for patients with arrhythmias, demonstrating reductions in cardiac anxiety, improvements in QoL, and decreased health care utilization, with mediation analyses identifying cardiac anxiety as a key mechanism of change.^29^ The protocol was subsequently adapted for patients after MI with elevated cardiac anxiety (CBT for cardiac anxiety [CA-CBT]). Two sequential pilot studies demonstrated feasibility and acceptability, with potential to improve clinically relevant outcomes.^18^

Key results are summarized in the concurrently published *JACC* Brief Report.^1^ This article provides the full methodological, architectural, and operational detail supporting those findings.

### Study objective

The present RCT evaluates the efficacy of digital, exposure-based CBT for cardiac anxiety compared with usual care (UC) in patients with clinically significant cardiac anxiety following MI. The primary clinical outcomes of disease-specific health status and cardiac anxiety at 3 months after treatment completion are reported in the companion Brief Report, *JACC*.^1^ This article describes the study design and methodology, and reports secondary outcomes, including depression, general anxiety, and fear of bodily sensations, objectively measured physical activity assessed by accelerometry, and cardiac-related biomarkers (eg, low-density lipoprotein [LDL] and hsCRP).

## Methods

### Study design

This RCT was conducted at Karolinska University Hospital in Stockholm, Sweden, with nationwide recruitment. Recruitment started on October 17, 2022 and ended on December 5, 2023, and final data collection was completed on March 17, 2025. The study was pre-registered at ClinicalTrials.gov (NCT05580718) and approved by the Swedish Ethical Review Authority (no 2022-04087-01). All participants provided informed consent. The trial was conducted in accordance with relevant regulations and the Declaration of Helsinki. The authors confirm adherence to the trial protocol and affirm that the data are complete and accurate.

### Participants

#### Eligibility criteria

The eligibility criteria for participation in the trial were as follows: 1) MI ≥6 months before assessment (type 1 ST-segment elevation myocardial infarction/non–ST-segment elevation myocardial infarction);^30^ 2) age 18 to 80 years; 3) clinically significant cardiac anxiety, defined by functional impairment and interference with daily life, as assessed via structured interview by a clinical psychologist; 4) medical care as deemed adequate by the study cardiologist; and 5) able to read and write in Swedish. Exclusion criteria were: 1) heart failure with reduced ejection fraction (≤35%); 2) significant valvular disease; 3) planned coronary artery bypass surgery or percutaneous interventions; 4) any medical restriction to physical exercise; 5) severe medical illness or any other condition that would preclude participation (eg, planned major surgery within 3 months or terminal illness); 6) grade 3 hypertension (ie, blood pressure ≥180 systolic and/or ≥110 diastolic); 7) severe mental illness according to ICD-11 criteria, including severe depression or risk of suicide^31^; 8) alcohol dependency; and 9) ongoing psychological treatment.

Participants in the trial continued their medical management post-MI as usual but were asked not to participate in any concurrent psychological treatment until CA-CBT had been concluded. The lower limit of 6 months after MI was set to ensure medical stability and an enduring presentation of cardiac anxiety. No upper time limit was applied; participants could have experienced their MI at any time before enrollment, as cardiac anxiety may persist after the index event.^32^^,^^18^

#### Recruitment and determination of eligibility

Participants were recruited from all over Sweden via self-referral in response to advertisement in social media and daily press, and information from health care providers/cardiology practices. Applicants registered on a secure web-based platform, provided online informed consent, and completed online screening questionnaires. The screening questionnaires included demographic questions, questions on their medical history, the Seattle Angina Questionnaire (SAQ),^33^ the Cardiac Anxiety Questionnaire (CAQ),^34^ the Patient Health Questionnaire-9 (PHQ-9),^35^ and the Alcohol Use Disorders Identification Test.^36^ The purpose of this initial screening was to inform the clinical interview and to flag potential safety concerns before further assessment.

Eligibility was determined through a two-step eligibility assessment combining medical and psychological evaluations to ensure participant safety, verify medical stability, and establish cardiac anxiety as the primary clinical focus, and exclude other psychiatric conditions requiring specialized treatment.

##### Stage 1: Medical eligibility assessment

A study nurse (E.Ó.) conducted the initial assessment; reviewed electronic medical records and medical history and conducted a structured telephone-based clinical interview to assess eligibility criteria 1-5 and exclusion criteria 1-6. The study nurse verified MI diagnoses, cardiac parameters (including ejection fraction), comorbid conditions, and current treatments to confirm medical stability and identify any contraindications to participation. Office blood pressure values from the preceding 3 months were confirmed through medical records; if unavailable, participants provided a recent office or home measurement. This review was facilitated by the National Patient Overview, a secure nationwide health information exchange in Sweden that allows authorized health care personnel to access patients’ electronic medical records across care providers when assessing medical eligibility.

##### Stage 2: Psychological eligibility assessment

Clinical psychologists (A.J., BE.L., and L.M.), conducted structured telephone-based clinical assessments to assess cardiac anxiety (criterion 3) and psychological exclusion criteria (criteria 7-9). Clinically significant cardiac anxiety was evaluated based on functional impairment and interference with daily life (eg, restricting activities due to cardiac-related worry), no questionnaire cutoff was applied. The clinical interview further confirmed that the anxiety was primarily cardiac specific rather than attributable to another primary psychiatric disorder. In addition, the interview ensured that participants did not present with severe mental illness according to ICD-11 (eg, severe depression), suicide risk, or alcohol dependence, as these conditions require specialized care beyond the scope of the trial. Comorbid anxiety disorders and moderate depression were permitted. Cardiac anxiety could occur alongside these conditions but was required to be the primary and clinically predominant concern for the inclusion in this trial.

##### Final medical clearance by study cardiologist

The study cardiologists (E.R. and L.G.M.) performed a final medical review of the clinical interviews and medical records to evaluate medical history, blood pressure, current pharmacological treatment, overall cardiac status, and to confirm the absence of contraindications to physical activity. This final assessment ensured medical eligibility and verified that no medical contraindications to participating in a therapist-guided psychological intervention were present before inclusion.

Excerpts from the study nurse assessment and psychologist interview guides, the study cardiologist checklist, a description of differential diagnostic considerations, anonymized examples of inclusion and exclusion decisions, and demographic characteristics of excluded participants are provided in the Supplemental Material.

### Trial procedures

Following inclusion, participants completed baseline assessments, including self-reported questionnaires, accelerometry, and blood sampling (detailed under Outcomes). Participants were randomized individually in a 1:1 ratio to CA-CBT or UC using a computer-generated random sequence (random.org). Simple randomization was performed sequentially at the time of inclusion by an external party, without prespecified blocks or stratification, maintaining allocation concealment and ensuring equal probability of assignment for each participant. Given the moderate sample size and the primary aim of estimating the overall treatment effect rather than subgroup effects, simple randomization was considered appropriate. The treatment started within 3 working days of randomization. Participants were informed that those randomized to UC would be offered CA-CBT after completing the 3-month follow-up.

Online self-reported assessments, accelerometer data, and blood samples were collected at baseline, post-treatment (8 weeks after baseline) and at 3-months post-treatment. The 3-month follow-up (primary end point) occurred approximately 5 months after baseline, corresponding to 3 months after completion of the 8-week CA-CBT intervention. The self-reported assessments were conducted online without involvement from the study personnel. Both arms also completed online self-reported assessments weekly throughout the 8-week treatment period. Participants randomized to CA-CBT received the intervention in addition to UC and completed the same weekly assessments as the UC group. The weekly data were collected for moderation and mediation analysis and are not reported in the present paper. In addition, the CA-CBT group completed a 12-month follow-up. The study nurse was blinded to treatment allocation, but due to the nature of the intervention, participants and therapists were not.

#### Intervention overview and rationale

CA-CBT is a tailored adaptation of exposure-based CBT for cardiac anxiety post-MI. The protocol was developed by Särnholm et al^26^, ^27^, ^28^ for cardiac anxiety in patients with atrial fibrillation and subsequently adapted and refined for patients post-MI through 2 sequential pilot studies demonstrating feasibility and acceptability.^18^

The intervention uses an exposure-based CBT approach targeting cardiac-specific fear, hypervigilance to cardiac-related sensations, and avoidance of activities after MI. Grounded in established exposure principles designed to activate fear responses, violate threat-related expectations, and promote inhibitory learning,^20^ CA-CBT applies these mechanisms to cardiac-related fears through structured interoceptive exposure (ie, deliberate activation of feared cardiac-related bodily sensations) and situational exposure targeting activities avoided after MI along with MI-specific psychoeducation. This cardiac-specific focus and disease-specific adaptation distinguish CA-CBT from standard CBT for generalized anxiety or panic disorder, which primarily targets diffuse worry or panic-related fear^37^ rather than disease-specific avoidance patterns.

#### Treatment components

CA-CBT included the following treatment components: 1) *Psychoeducation* addressing cardiac anxiety, avoidance behaviors, and its effect on health behaviors and QoL, combined with individualized goal setting, behavioral analysis of a situations eliciting cardiac anxiety, and mapping of post-MI avoidance behaviors. This component also included education on benign vs acute cardiac symptoms and guidance on when to seek medical attention; 2) *Labeling* (ie, identifying and naming cardiac-related sensations, behavioral impulses, emotions, and thoughts) to enhance awareness and build tolerance of bodily sensations and emotional responses; 3) *Exposure to cardiac-related bodily sensations* (interoceptive) aimed to reduce fear and hypervigilance toward cardiac-related sensations. Exercises were selected to safely evoke bodily sensations commonly associated with cardiac anxiety, such as elevated heart rate or shortness of breath. Examples included engaging in physical activity, lying on the left side to increase awareness of heartbeats, and controlled overbreathing; 4) *Situational exposure* (in-vivo) targeting avoidance of situations and activities such as physical exercise, travel beyond familiar environments, planning for the future, and engagement in daily life activities. Individual exposure tasks were tailored to participant’s mapped post-MI avoidance behaviors, which were tracked throughout treatment; 5) *Reduction of control and safety behaviors*, including reduction of continuous heart monitoring, frequent pulse checking, and heightened situational awareness related to proximity or location. The treatment components were applied in combination and tailored to each participant; for example, a brisk walk while practicing labeling sensations and refraining from pulse checking; and 6) *Relapse prevention* focused on planning for continued engagement in treatment strategies and managing potentially challenging situations.

Module content descriptions and selected excerpts of the treatment content are provided in the Supplemental Material as well as in Johnsson et al.^18^

#### Treatment delivery and therapist support

The intervention was therapist guided, delivered digitally over 8 weeks via a secure web-based platform and consisted of 8 interactive, text-based modules. Participants completed weekly homework and exposure exercises, reported progress on the platform, and received asynchronous, individualized text-based therapist feedback within 48 hours. Therapist guidance was provided by 2 licensed clinical psychologists (A.J. and B.E.L.) and a resident clinical psychologist under supervision (L.M.). Each module required approximately 30 to 60 minutes for participants to complete, and participants were encouraged to allocate approximately 20 minutes per day engaging in exposure exercises in their daily lives. Modules were made available based on participant progress, following therapist confirmation of completion.

Fidelity checks were conducted weekly by the lead psychologists within the web-based platform to ensure adherence to the treatment protocol, confirm consistency with the intervention rationale, and verify that no nonprotocol elements were introduced. Fidelity checks did not indicate any deviations from the treatment protocol.

#### Multidisciplinary collaboration and participant safety

Before treatment delivery, study psychologists received training in MI and general post-MI care from the study cardiologists (L.G.M. and E.R.) and the study nurse (E.Ó.) and protocol-specific training in CA-CBT from the senior author (J.S.). Training included participant safety procedures for managing cardiac symptoms, with emphasis on distinguishing benign from acute cardiac symptoms and guidance on adapting exposure tasks to individual clinical profiles. Psychologists were instructed in escalation protocols and in monitoring symptom reports during exposure exercises, with instructions to pause or modify tasks if new, unexpected, or concerning symptoms occurred.

During treatment delivery, psychologists had on-demand supervision by lead psychologists (A.J. and J.S.) and access to clinical consultation from the study nurse and cardiologists via the web-based platform for clinical concerns. This structure enabled individualized supervision on request and facilitated active collaboration when clinical judgment was required, ensuring that exposure exercises were conducted safely and were medically appropriate.

Participants received clear instructions on when to discontinue exposure exercises and were educated on differentiating benign cardiac-related sensations from symptoms requiring medical attention. Examples of benign symptoms included transient palpitations, mild exertional shortness of breath, and brief chest discomfort without progression. Symptoms warranting immediate medical contact included persistent or worsening chest pain or pressure, particularly when radiating to the arm or jaw. Guidance on when to seek emergency or primary care was incorporated into the treatment materials.

Excerpts of instructions for psychologists and education to participants are provided in the Supplemental Material.

#### Usual care

The UC-group completed the full baseline, post-treatment, and 3-month follow-up assessments. Weekly during the 8 week treatment period, participants completed weekly online self-assessments comprising the SAQ,^33^ the CAQ,^34^ the Perceived Stress Scale (PSS-4),^38^ and adverse events.^22^ Health care utilization in the preceding 4 weeks was collected using the TIC-P A-section,^39^ at baseline, post-treatment, and 3-month follow-up for both groups and is reported descriptively in this study to contextualize the comparison condition.

UC following MI in Sweden usually include cardiac rehabilitation (CR) in tertiary care during the first post-MI year, involving regular contact with cardiac nurse, physiotherapist, and cardiologist.^40^ After the first year, clinically stable patients are typically transitioned to primary care for ongoing management and long-term secondary prevention; the specific content and intensity of follow-up vary. Participants were instructed to follow the advice and care from their health care providers during the trial. UC did not include any structured psychological or behavioral cardiac intervention.

### Outcomes

A description of outcomes is provided subsequently, with further information on secondary outcomes available in the Supplemental Material. Outcomes were assessed at baseline, at post-treatment (8 weeks after baseline), at 3-month-follow-up (5 months after baseline; primary end point), and at 12-month follow-up (intervention group only).

#### Primary end point

The SAQ is a disease-specific patient-reported outcome measure consisting of 19 items across 5 subscales: QoL, physical limitation, angina frequency, angina stability, and treatment satisfaction.^41^ The SAQ does not yield a summary score; each subscale is scored from 0 to 100, with higher scores indicating better disease-specific health status.^33^ The QoL and physical limitation subscales can additionally be categorized as poor, fair, good, or excellent health status.^42^ The primary end point for this trial was the between-group difference in the estimated mean change in disease-specific health status from baseline to the 3-month follow-up, assessed using 4 subscales of the SAQ: QoL, physical limitations, angina frequency, and angina stability.^41^ The treatment satisfaction subscale (items 5-8) was not analyzed, as it reflects satisfaction with medical treatment, which is not a scope of the present study.

The SAQ was selected as the primary end point because it captures dimensions of disease-specific health status, a patient-centered outcome prioritized in cardiovascular care.^42^ SAQ is widely used in trials and registries of coronary artery disease and endorsed as a validated patient-reported outcome with prognostic importance and sensitivity to clinical change. Its use enables benchmarking against other post-MI interventions and reflects the broader impact of psychological distress on functioning and recovery. The primary end point results are presented in the companion Brief Report in *JACC*.^1^

#### Self-reported secondary outcomes

Self-reported secondary outcomes included cardiac anxiety (CAQ),^34^ fear of bodily symptoms (Body Sensation Questionnaire [BSQ]),^43^ cardiac-related symptoms: (Symptom Checklist Severity and Frequency Scale),^44^ physical activity (The Godin Leisure-time Exercise Questionnaire),^45^ depressive symptoms (PHQ-9),^46^ generalized anxiety (Generalized Anxiety Disorder −7 [GAD-7]),^47^ perceived stress (PSS-4),^38^ health-related QoL (12 Short-Form Health Survey [SF-12]),^48^ health care seeking (3 items from University of Toronto Atrial Fibrillation Severity Scale [AFSS]^49^). Detailed descriptions are provided in the Supplemental Material.

#### Physical activity behaviors measured by accelerometry

Physical activity was assessed at baseline, post-treatment, and 3-month follow-up using accelerometry. Participants wore an ActiGraph (model wGT3X-BT) on their nondominant wrist for 7 consecutive 24 h-periods. Further details are provided in the Supplemental Material.

#### Cardiac-related biomarkers

Fastening blood samples were collected at baseline, post-treatment, and 3-month follow-up to assess metabolic, lipid, inflammatory, and renal biomarkers. A total of 20 mL of fasting venous blood was drawn at each time. Measurements included average blood glucose levels (hemoglobin A1c), total cholesterol (TC) levels, atherogenic cholesterol (LDL), antiatherogenic cholesterol (high-density lipoprotein), lipids (triglycerides), inflammation markers (high-sensitivity C-reactive protein [hsCRP]), and kidney function (serum creatinine). The selected biomarkers reflect prevention targets (e.g., LDL, hemoglobin A1c) and were analyzed to examine whether the intervention could influence these variables by impacting behaviors such as adherence to lifestyle recommendations (eg, dietary advice, adherence with drug treatment) and reducing anxiety. Creatinine and hsCRP were measured as markers of renal function and systemic inflammation, respectively, reflecting overall disease burden and comorbidities. Given the conflicting evidence for direct effects of psychological interventions on cardiovascular biomarkers,^50^ these analyses were intended to provide additional context on potential physiological correlates. Further details are provided in the Supplemental Material.

#### Treatment activity, adherence, and satisfaction with the treatment

Adherence was assessed based on the number of completed modules and psychologist monitoring of behavior logs, exposure exercises, and weekly homework. Modules were released sequentially based on therapist-confirmed completion, and the total number of successfully completed modules was documented at treatment end. CA-CBT treatment satisfaction was assessed in the intervention group using the Client Satisfaction Questionnaire (CSQ-8).^51^ It includes 8 questions, with scores ranging from 8 to 32, where higher scores reflect greater satisfaction. In addition, the final module included open-ended questions inviting participants to describe their treatment experience.

#### Adverse events

Adverse events were collected weekly during the 8 week intervention period, at post-treatment and follow-up using a structured, patient-reported questionnaire.^22^ Participants were asked, “Have you experienced any unwanted event or unwanted effect during the treatment that you believe was caused by the treatment?” Participants who responded “yes” provided a free-text description detailing when the event occurred, how often it occurred, how long it lasted, and its characteristics. They then rated the negative impact of the event on a four-point scale ranging from “Did not affect me at all” to “Affected me very negatively”. Participants could report up to 3 adverse events within the structured items, and an additional open-ended field allowed reporting of any further events.

### Power calculation

The sample size was informed by effect estimates from our previous RCT of digitally delivered exposure-based CBT in patients with atrial fibrillation, which used atrial fibrillation effect on quality-of-life (AFEQT)^26^ as primary outcome. AFEQT is conceptually similar to SAQ, a disease-specific disability and cardiac symptoms outcome. Because the post-MI pilot studies^18^ preceding the RCT used non disease-specific measures of health status (SF-12), they did not inform the present calculation. Given differences in patient populations and outcome instruments, we conservatively assumed a between-group effect size of d = 0.60 for change in SAQ subscale scores at 3 months. This effect size required a total sample size of 100 participants to achieve 80% power, allowing for 10% attrition. Given the high retention rate observed in the present study (4% lost at 3-month follow-up), recruitment was concluded at 96. Notably, the power calculation was based on single-outcome statistics and did not take the use of multiple correlated subscales, with subsequent false discovery rate (FDR) adjustment, into account.

### Statistical analysis

For the outcome analyses, we primarily used hierarchical linear mixed models with a random intercept to account for within-individual dependence in data. In accordance with the intention-to-treat principle, all available randomized participants were included in the analyses at post-treatment and 3-month follow-up. Mixed-effects models accommodate missing observations under the assumption of missing at random. No imputation or other methods for handling missing data were applied, given the low attrition rate at the primary end point (4%). The dependent variable was the raw score of each individual at the 3 time points baseline, post-treatment, and 3-month follow-up. The independent variables (fixed effects) were group, time, and the group∗time interaction effects. Time was coded as a three-level factor (baseline, post-treatment, and 3-month follow-up) with baseline as the reference value. Thus, we simultaneously modeled the average change from baseline to post-treatment and change from baseline to 3-month follow-up. The 2 interaction effects produced by the model, group∗time(post-treatment) and group∗time(3-month follow-up), estimated the difference between the groups in change from baseline to the respective end point (post-treatment and 3-month follow-up) and thus tested the hypothesis that the CA-CBT group would show greater improvements across time points compared to the UC group. *P* values obtained from 3-month follow-up contrasts were corrected for multiple tests using the FDR correction,^52^ separately for the self-assessed outcomes (18 *P* values) and the exploratory analyses of cardiovascular biomarker data and accelerometer data (8 *P* values).

Analytical procedures related to the SAQ, cardiac health care visits, accelerometer data, and 12-month follow-up data as well as statistical software used are described in the Supplemental Material. As sensitivity analyses, we also conducted a set of analysis of variance analyses in which we controlled for baseline values of the outcome of interest and examined if baseline characteristics moderated the treatment effects. These analyses are further described in the Supplemental Material.

## Results

### Recruitment sources

Participants were recruited nationwide through multiple channels, most commonly via social media (40%), information from health care providers (30%), and newspaper advertisements (16%), as well as other nonspecified sources, including self-initiated online searches (14%).

### Participant flow

**Figure 1:**
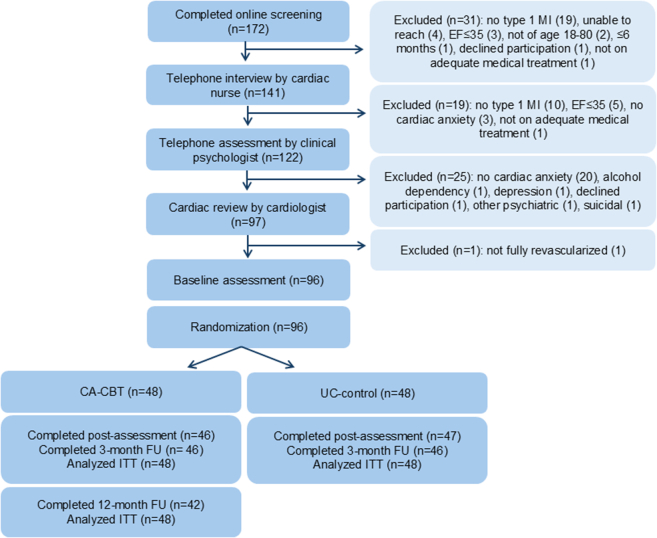
**Flow of Participants Through the Study** This flowchart illustrates the process of assessment of eligibility and follow-up in the study. The flowchart is divided into 2 main sections: assessments of participants and randomization and follow-up. The assessment section also outlines the exclusion criteria. The follow-up section outlines the number of participants completing the follow-up assessments. Arrows and boxes are used to indicate the flow of patients through the study. CA-CBT = cognitive behavioral therapy for cardiac anxiety; EF = ejection fraction; FU = follow-up; ITT = intention-to-treat; MI = myocardial infarction; UC = usual care.

The participant flow including the numbers and reasons for exclusion are shown in Figure 1. Of the 172 individuals screened online, 31 were excluded due to ineligibility. A total of 141 underwent a telephone interview with the study cardiac nurse and 19 were excluded. Of the remaining 122, structured telephone assessment by a clinical psychologist led to the exclusion of 25. Ninety-seven participants underwent cardiologist review; 1 was excluded for not being fully revascularized. The final sample comprised 96 participants who completed baseline assessment and were randomized to CA-CBT (n = 48) or UC (n = 48). Demographic characteristics (age, sex, education, and employment status) of excluded participants are provided in Supplemental Table 1.

### Sample

The final sample included 96 participants (median age 65.0, IQR: 56.5-70.1, 73% male, median time since MI 2.2 years, IQR: 1.1-4.9), with 48 in the CA-CBT group and 48 in the UC group. The mean score on the SAQ QoL subscale (44.8 ± 19.4) indicated fair levels,^42^ and the average CAQ score indicated clinically significant cardiac anxiety (31.3 ± 9.3).^32^ No clinically relevant differences were observed, although the CA-CBT group had a slightly higher proportion of retired participants (Table 1), which was addressed in sensitivity analyses (see Sensitivity analyses given subsequently).

**Table 1:** Participants Characteristics at Baseline

	All (N = 96)	CA-CBT (n = 48)	UC (n = 48)
Age, y	65.0 (56.5-70.1)	66.1 (58.7-71.9)	62.2 (55.7-69.2)
Sex			
Female	26 (27%)	14 (29%)	12 (25%)
Male	70 (73%)	34 (71%)	36 (75%)
Occupation			
Employed	55 (57%)	23 (48%)	32 (67%)
Retired	36 (38%)	21 (44%)	15 (31%)
Unemployed	1 (1%)	1 (2%)	0 (0%)
Sick leave	3 (3%)	2 (4%)	1 (2%)
Student	1 (1%)	1 (2%)	0 (0%)
Highest completed education			
Less than high school	5 (5%)	4 (8%)	1 (2%)
High school	19 (20%)	8 (17%)	11 (23%)
Post-high school, not university	17 (18%)	10 (21%)	7 (15%)
University	55 (57%)	26 (54%)	29 (60%)
Medical history			
Hypertension	57 (59%)	28 (58%)	29 (60%)
Diabetes	12 (13%)	5 (10%)	7 (16%)
Obstructive sleep apnea	13 (14%)	6 (13%)	7 (15%)
Smoking	10 (10%)	2 (4%)	8 (17%)
Angina pectoris	6 (6%)	4 (8%)	2 (4%)
Atrial fibrillation	10 (10%)	4 (8%)	6 (13%)
Previous stroke	4 (3%)	3 (6%)	1 (2%)
Previous MI	7 (7%)	5 (10%)	2 (4%)
Previous cardiac rehab	50 (52%)	27 (56%)	23 (48%)
Most recent MI			
Yr since MI	2.2 (1.1-4.9)	1.5 (1.0-4.2)	2.8 (1.5-5.1)
STEMI	47 (48%)	25 (52%)	22 (46%)
N/STEMI	49 (52%)	23 (48%)	26 (54%)
PCI	81 (84%)	42 (88%)	39 (81%)
CABG	13 (14%)	6 (13%)	7 (15%)
Conservative treatment	2 (2%)	0 (0%)	2 (4%)
Mental health status^a^			
Depressed mood	28 (29%)	15 (31%)	13 (27%)
Any anxiety disorder	33 (34%)	15 (31%)	18 (38%)
Insomnia	38 (40%)	18 (38%)	20 (42%)
Physical and Cardiovascular Status			
BMI	26.3 (24.6-28.7)	26.5 (24.8-28.5)	26.2 (24.1-28.7)
Blood pressure systolic, mm Hg	125.0 (117.5-130.5)	122.0 (117.0-130.0)	127.0 (118.5-131)
Blood pressure diastolic, mm Hg	75.0 (68.5-80.0)	75.0 (67.5-80.0)	74.5 (69.0-80.0)
Heart rate, beats/min	64.0 (57.0-69.0)	63.5 (58.0-69.0)	64.0 (55.0-70.0)
EF ≤40%	8 (8%)	3 (6%)	5 (10%)
EF 41%-49%	10 (10%)	8 (17%)	2 (4%)
EF ≥50%	78 (81%)	37 (77%)	41 (85%)
MVPA (min/day)	31.6 (17.3-51.9)	29.0 (14.3-51.8)	36.5 (22.5-52.9)
Current medication			
Beta blockers	64 (67%)	35 (73%)	29 (60%)
ACEi/ARB	75 (78%)	37 (77%)	38 (79%)
Calcium channel blocker	23 (24%)	12 (25%)	11 (23%)
Lipid-lowering agents	94 (98%)	47 (98%)	47 (98%)
ASA/DAPT	83 (86%)	43 (90%)	40 (83%)
NOAC/Warfarin	13 (14%)	5 (10%)	8 (17%)
Antidepressants	10 (10%)	7 (15%)	3 (6%)
Sleep medication	4 (4%)	1 (2%)	3 (6%)
Anxiolytics, ”as needed”	3 (3%)	3 (6%)	0 (0%)

Values are as median (IQR) or n (%). Occupation categories are mutually exclusive and reflect participants’ primary activity (employed, unemployed, retired, student, sick leave).

ACEi = angiotensin-converting enzyme inhibitor; ARB = angiotensin receptor blocker; ASA = acetylsalicylic acid; BMI = body mass index; Bpm = beats per minute; CA-CBT = CBT for cardiac anxiety; CABG = coronary artery bypass graft surgery; DAPT = dual antiplatelet therapy; EF = ejection fraction; MI = myocardial infarction; MVPA = moderate-to-vigorous physical activity; NOAC = non-vitamin K antagonist oral anticoagulant; N/STEMI = non–ST-segment elevation myocardial infarction; PCI = percutaneous coronary intervention; STEMI = ST-segment elevation myocardial infarction; UC = usual care.

a Mental health status as assessed by study clinical psychologist.

At post-treatment, 2/48 (4%) in the CA-CBT group and 1/48 (2%) in the UC group were lost to follow-up. In the CA-CBT group, the reasons for loss to follow-up included psychosocial stressors and an eye-related medical condition. By the 3-month follow-up (primary end point), a total of 4/96 (4%) participants were lost across both groups (2/48 [4%] per group). At 12 months, attrition in the CA-CBT group was 6/48 (13%). Given the limited attrition at the primary end point (4%), we did not impute missing data. Participant flow through the study is presented in Figure 1.

### Usual care context

Post-MI care in Sweden typically includes access to structured CR during the first year through a national program (based on the national registries SEPHIA/SWEDEHEART), with a standardized follow-up framework in cardiology or internal medicine clinics, including systematic evaluation of treatment, rehabilitation, and risk-factor management.^40^ After 1 year, clinically stable patients are typically transitioned to primary care for ongoing management and follow-up, which may involve general practitioners as well as other primary care professionals, such as nurses and physiotherapists. In this sample, most participants were more than 1 year post-MI at baseline (median 1.5 years in CA-CBT and 2.8 in UC). Overall, 52% reported having completed CR before study entry (56% in CA-CBT; 48% in UC).

Health care visits were collected at post-treatment and 3 months (section A, TIC-P). Patterns of health care visits included visits to cardiologists, general practitioners, nurses, and physiotherapists; reflecting care as usual post-MI. No participant reported receiving structured psychological treatment during the study period. Cardiac-specific health care utilization assessed with the AFSS is reported in Table 2. Additional details are provided in the Supplemental Material.

**Table 2:** Estimated Between-Group Differences in Mean Change at Post-treatment (Eight Weeks) and at 3-Month Follow-Up

Outcome	Raw Score at Baseline		Raw Score at Post-Treatment		Raw Score at 3-Month Follow-Up		Least Square Mean Change from Baseline to 3-Month Follow-Up		Est Diff in Mean Change Baseline to 3-Month Follow-Up	*P* Value	
	CA-CBT (n = 48)	UC (n = 48)	CA-CBT (n = 46)	UC (n = 47)	CA-CBT (n = 46)	UC (n = 46)	CA-CBT	UC		Raw	FDR
SAQ QoL	45.8 ± 21.7	43.8 ± 16.9	65.0 ± 20.3	51.8 ± 21.7	65.9 ± 24.3	53.8 ± 21.1	19.9 ± 2.4	9.5 ± 2.4	10.3 (3.6-17.0)	0.003	0.021
SAQ Physical^a^	82.8 ± 20.3	81.3 ± 17.4	91.3 ± 15.7	82.3 ± 16.5	90.8 ± 16.0	84.4 ± 17.0	7.4 ± 1.4	2.1 ± 1.4	5.2 (1.4-9.1)	0.007	0.028
SAQ Stability	81.2 ± 26.5	75.0 ± 25.3	91.8 ± 21.8	73.9 ± 27.6	89.1 ± 24.0	78.8 ± 25.3	7.8 ± 4.4	3.1 ± 4.4	4.7 (−7.5-16.9)	0.449	0.561
SAQ Frequency	85.6 ± 16.9	82.9 ± 15.0	89.8 ± 15.1	82.8 ± 16.3	91.7 ± 14.2	87.0 ± 15.0	6.2 ± 2.1	3.2 ± 2.1	3.1 (−2.8-8.9)	0.299	0.471
CAQ	30.1 ± 9.7	32.5 ± 8.7	16.0 ± 9.1	27.9 ± 8.5	17.3 ± 8.7	27.8 ± 8.2	−12.5 ± 1.0	−4.7 ± 0.9	−7.8 (−10.5 to −5.2)	<0.001	<0.001
PHQ-9	7.8 ± 6.0	7.9 ± 4.9	4.9 ± 4.6	6.9 ± 3.8	4.9 ± 5.3^b^	6.7 ± 4.2	−2.8 ± 0.5	−1.1 ± 0.5	−1.7 (−3.2 to −0.2)	0.025	0.074
GAD-7	6.1 ± 4.7	6.9 ± 4.9	3.9 ± 3.8^b^	5.2 ± 3.4	3.4 ± 3.3^b^	5.5 ± 3.9	−2.9 ± 0.5	−1.3 ± 0.5	−1.6 (−3.0 to −0.2)	0.030	0.074
BSQ	36.5 ± 10.5	40.5 ± 11.7	27.0 ± 6.0^b^	37.9 ± 10.5	26.9 ± 7.7^b^	36.0 ± 2.3	−9.8 ± 1.3	−4.8 ± 1.3	−5.1 (−8.8 to −1.4)	0.007	0.028
GLTEQ	71.4 ± 107.4	48.0 ± 45.9	82.6 ± 131.5^b^	51.4 ± 82.8	64.0 ± 91.3^b^	68.0 ± 132.8	−8.0 ± 19.4	19.6 ± 19.3	−27.6 (−81.6-26.4)	0.314	0.471
AFSS	0.8 ± 1.0	0.7 ± 1.8	0.5 ± 0.9^b^	0.7 ± 1.5	0.5 ± 0.8^b^	0.5 ± 1.5	0.8 (0.5-1.4)	0.6 (0.3-1.0)	0.7 (0.3-1.5)	0.400	0.546

Raw scores are presented as mean ± SD. Changes in least-square means from baseline to the 3-month follow-up (primary end point) and the estimated difference at 3 months were derived from linear regression mixed models. The least-square mean changes are shown as estimated change ± SE. The estimated differences are presented with 95% CIs. AFSS (health care visits) analyses are based on Poisson generalized linear mixed models with a log-link function. Estimates are presented as percent change/difference with 95% CIs.

AFSS = Toronto Atrial fibrillation Severity Scale; BSQ = Body Sensation Questionnaire; CAQ = Cardiac Anxiety Questionnaire; FDR = *P* value correct for the False Discovery Rate; GAD-7 = Generalized Anxiety Disorder 7-item scale; GLTEQ = The Godin Leisure-time Exercise Questionnaire; PHQ-9 = Patient Health Questionnaire; QoL = Quality of Life; SAQ = Seattle Angina Questionnaire; other abbreviations as in Table 1.

a One outlying score removed at post-treatment assessment.

b (n = 45) 1 participant provided partial data due to perceived assessment burden.

### Main outcomes

The primary end point results and their clinical implications are presented in the companion Brief Report, *JACC*.^1^ In summary, we observed statistically significant improvements in disease-specific health status on the SAQ QoL and physical limitation subscales, as well as significant reductions in cardiac anxiety, demonstrated by improvements on the CAQ total score and all three CAQ subscales. No significant changes were observed for SAQ angina stability or frequency subscales. Within-group results are displayed in Supplemental Table 4, and Cohen’s d effect sizes in Supplemental Table 6.

### Secondary outcomes

#### Self-reported secondary outcomes

At 3-month follow-up, CA-CBT was associated with significant reductions in fear of bodily sensations compared with UC. The estimated between-group difference in the mean change on the BSQ was −5.1 points (95% CI: −8.8 to −1.4; FDR-adjusted *P* = 0.028) (Table 2). Baseline average fear of bodily sensations scores (BSQ: 36.5) were elevated relative to normative values for nonclinical samples.^43^ Baseline average depressive symptoms (PHQ-9: mean 7.85) and general anxiety (GAD-7: mean 6.5) were in the mild range based on established norms.^35^^,^^47^ Although depressive symptoms and general anxiety initially showed modest between-group differences, the estimated mean differences at 3 months did not remain statistically significant after correction for multiple testing. For PHQ-9, the estimated difference was −1.7 points (95% CI: −3.2 to −0.2; FDR-adjusted *P* = 0.057), and for GAD-7, the estimated difference was −1.6 points (95% CI: −3.0 to −0.2; FDR-adjusted *P* = 0.059).

No between-group differences were observed for self-rated physical activity (Godin Leisure-time Exercise Questionnaire) or cardiac-related health care visits (AFSS), with the estimated mean differences reported in Table 2. Additional self-reported secondary outcomes (Symptom checklist Severity and Frequency Scale, SF-12, and PSS-4) likewise showed no between-group differences, with effects reported in Supplemental Table 5. Within-group improvements in the CA-CBT group were maintained at the 12-month follow-up (Supplemental Table 5), and between- and within-group effect sizes are reported in Supplemental Table 6.

#### Sensitivity analyses

Analyses of variance that predicted 3-month follow-up scores from group and included the outcome of interest as a covariate, replicated the main findings, that is, the effect of group on SAQ QoL (difference 10.7 points; 95% CI: 3.2-18.3; *P* = 0.006), SAQ physical limitation (difference 5.3 points; 95% CI: 1.8-8.9; *P* = 0.004), and CAQ (difference 8.8; 95% CI: 6.1-11.5; *P* < 0.001). None of the candidate moderators, age (all Ps > 0.08), years since MI (all *P* > 0.53), employment status (all *P* > 0.26), or CAQ baseline score (all *P* > 0.62), showed a significant interaction effect with group, meaning that there was no evidence that the evaluated baseline characteristics affected treatment effects.

#### Physical activity behaviors measured by accelerometry

At baseline, the median daily moderate to vigorous physical activity was 29.0 minutes/day (IQR: 14.3-51.8) in the CA-CBT group and 36.5 minutes/day (IQR: 22.5-52.9) in the UC group, corresponding to overall activity levels within the range recommended by guidelines.^53^ At the 3-month follow-up, moderate to vigorous physical activity levels remained similar across group. The mean change from baseline, −4.7 ± 3.0 minutes/day in CA-CBT vs −4.4 ± 2.8 minutes/day in UC, yielded an estimated between-group difference of −0.3 (95% CI: -8.4-7.7). No between-group differences were observed in light physical activity, with mean changes of 2.6 ± 5.3 minutes/day vs 4.9 ± 4.9 minutes/day, corresponding to an estimated difference of −2.3 (95% CI: -16.6-12.0). Similarly, changes in sedentary time did not differ between groups. Sedentary time decreased by −20.3 ± 10.3 minutes/day in CA-CBT vs −4.5 ± 9.6 in UC, corresponding to an estimated between-group difference of −15.7 (95% CI: −43.6 to 12.1). Supplemental Table 3 displays the estimated mean differences and *P* values of the accelerometer data.

#### Cardiac-related biomarkers

At baseline, the mean values of cardiac-related biomarkers met, or were close to, the target thresholds defined by clinical guidelines for patients with a previous MI (eg, LDL <1.4 mmol/L).^53^ Across the 3-month follow-up, no between-group differences were observed. For example, the estimated between-group difference in mean change for TC was −0.2 mmol/L (95% CI: −0.5 to 0.1). The estimated mean differences for all biomarkers are presented in Supplemental Table 4.

#### Treatment activity, adherence, and satisfaction with the treatment

A large proportion of participants in the CA-CBT group were classified as treatment completers (38/48; 79%), defined as having completed at least 4 modules and thus received the core components of the intervention. On average, participants completed 5.9 ± 2.4 of the 8 treatment modules. Treatment satisfaction was high, with a mean CSQ-8^51^ score of 25.7 ± 5.4 (maximum score: 32). Among the 45 participants who completed the CSQ-8 at post-treatment, 98% (44/45) rated the overall quality of the treatment as fair, good, or excellent. At treatment completion, participants’ reflections in response to open-ended questions in the final module, summarizing the treatment experience, highlighted the following themes: increased trust and confidence in the body and heart (eg improved differentiation of bodily sensations; engaging in physical activity with less worry), expanded strategies to manage cardiac anxiety through labeling and exposure (eg reduced threat perception; greater willingness to engage with emotions and take on new challenges; increased autonomy and social activities), and greater trust and confidence in the future (eg making plans).

#### Adverse events

There were no serious medical or cardiac events reported. Most reported events in both groups were mild and transient. In the CA-CBT group, the events were primarily related to stress due to study participation or heightened attention to cardiac-related sensations. During the 8-week treatment, 9 participants (19%) reported eleven such events. At post-treatment, 7 participants (16%) reported 8 adverse events, with none reported at the 3-month follow-up. In the UC group, most events were attributed to stress related to self-reported assessments and increased attention toward cardiac-related sensations. During the 8-week assessment period, 6 participants (13%) reported 7 adverse events and 5 participants (11%) reported 1 event each at post-treatment. One event was reported at the 3-month follow-up.

#### Changes in cardiac health and medication

At the 3-month follow-up, 5 participants (10%) in the CA-CBT group and 7 participants (15%) in the UC group self-reported perceived deterioration in cardiac health. The most reported concerns included increased symptoms, such as fatigue, chest tightness, and a perceived reduction in stamina. Changes in medication and other cardiac-related procedures were minimal. See Supplemental Material for detailed description.

## Discussion

Main results and their clinical implications are discussed in the paired Brief Report in *JACC*,^1^ whereas study design, methods, and secondary outcomes are discussed in the present paper.

This RCT evaluated the efficacy of a therapist-guided, digital exposure–based CBT intervention for cardiac anxiety after MI compared with UC. The intervention demonstrated high adherence and patient satisfaction and was delivered safely, with no serious adverse events observed. At 3-months (primary end point), participants receiving CA-CBT showed greater improvements in aspects of disease-specific health status, specifically on the SAQ QoL and physical limitation subscales, whereas no effects were observed on SAQ angina stability and angina frequency. We also observed reductions in cardiac anxiety and fear of bodily sensations, favoring CA-CBT. The within-group effects in the CA-CBT group were maintained at 12 months. Effects on depressive symptoms and general anxiety did not remain statistically significant after multiplicity adjustment. No between-group differences were observed in objective physical activity or cardiac-related biomarkers.

### Treatment adherence, delivery, and safety

Treatment satisfaction and treatment completion were high (79%); fidelity checks indicated protocol consistent delivery and attrition rates were low. Reported adverse events were mild and transient, most commonly stress and heightened attention to bodily sensations, and consistent with findings from prior exposure-based CBT in atrial fibrillation and other somatic conditions.^22^^,^^26^ The results described previously are likely attributable to several interrelated design features that aligned the intervention with patients’ clinical needs while supporting safe and consistent delivery. These included: 1) a structured psychological assessment confirming the presence of clinically significant cardiac anxiety to match the target of treatment and an individualized behavioral analysis that ensured tailored exposure tasks to the patient’s avoidance behaviors; 2) a digital, structured, and manualized treatment format and with asynchronous therapist guidance, supporting both treatment fidelity and tailoring to the patients’ clinical presentation; and 3) a multidisciplinary framework, including on-demand clinical consultation with study nurse and cardiologist, that conveyed medical safety and helped psychologists to support full engagement in exposure exercises.

Exposure-based interventions remain underused in medically vulnerable populations, largely due to concerns regarding safety.^21^^,^^23^ By embedding exposure within a multidisciplinary safety framework, this trial illustrates a pragmatic model for addressing these concerns and aligns with broader calls to strengthen multidisciplinary collaboration and training to integrate behavioral medicine into cardiovascular care.^16^^,^^54^

### Behavioral mechanistic rationale

The CA-CBT intervention addresses fear-driven hypervigilance and avoidance that lead patients to restrict physical and daily activities after MI.^32^^,^^8^^,^^10^ Through interoceptive and situational exposure, participants repeatedly engage with feared cardiac-related sensations and activities in a systematic manner. This process supports inhibitory learning, reduce threat expectations, increase confidence in bodily sensations, and enable re-engagement in physical and social activities. Consistent with this mechanism, mediation findings in atrial fibrillation suggest that using an exposure-based strategy to reduce cardiac anxiety are key drivers of improvements in QoL.^19^ This is also reflected in participants’ accounts of reinterpretation of bodily sensations, accompanied by a broadened behavioral repertoire and re-engagement in daily activities.

### Self-reported secondary outcomes

In addition to the primary clinical outcomes, reductions in fear of bodily sensations (BSQ) favored the CA-CBT group. In contrast, initial signals for depressive symptoms (PHQ-9) and general anxiety (GAD-7) did not remain statistically significant after FDR correction, potentially reflecting subclinical baseline symptom levels, limited power for secondary end points, and the conservative nature of multiplicity adjustment.

### Physical activity and cardiac-related biomarkers

Objective physical activity and cardiac-related biomarkers did not differ between groups, consistent with baseline values near guideline targets (ceiling effects), the relatively short follow-up window for physiological change, and sample size constraints. Small, nonsignificant trends toward reduced sedentary time and lower TC merit exploration in higher-risk cohorts. Health care utilization likewise did not differ between groups, plausibly reflecting low baseline use, exclusion of patients scheduled for procedures, medical activity restrictions, and a stable post-MI phase (floor effects), in contrast to atrial fibrillation populations with more frequent symptom fluctuations.^26^

### Study strengths and limitations

Key strengths of this study include a mechanistic behavioral rationale with a theory-informed behavioral target, thorough inclusion-procedure and multidisciplinary collaboration, the use of well-validated instruments, and the combination of both subjective (e.g., SAQ and CAQ) and objective measures (accelerometry and biomarkers). Furthermore, the nationwide recruitment enabled participation from patients regardless of geographic location, illustrating the potential for centralized delivery of exposure-based CBT that is currently not available in routine care.

The demographic and clinical profile of our sample (median age 65.0, IQR: 56.5-70.1, 73% male) is broadly comparable to large post-MI cohorts such as PREMIER (n ≈ 2,500; mean age ∼61 years; ∼70% male)^55^ and TRIUMPH (n ≈ 4,300; mean age ∼59 years; ∼70% male),^56^ which report similar age and sex distributions. The median time since MI in our sample (approximately 2 years) indicates that participants were in a stable phase, minimizing confounding from acute recovery. Sensitivity analyses showed that time since MI did not affect treatment outcomes. The broad range in time since MI also suggests that cardiac anxiety can persist over time and does not spontaneously remit.

Several limitations should be considered. The use of UC as a control condition did not account for therapist attention, structured engagement, or expectancy effects. However, participants in the UC group completed weekly self-reported assessments, potentially providing a form of structured attention and self-monitoring, also reflected in the modest improvements in the UC group. Participants in the UC group were aware of crossover access to CA-CBT after 3 months, which may have influenced expectancy and self-reported outcomes during the waiting period.

Notably, participants were medically stable; many had previously completed CR and had internet access, suggesting a cohort that may be more health conscious and digitally literate than the broader post-MI population. Self-referral may also have introduced selection bias toward motivated individuals. These factors should be considered when interpreting external validity, and future studies should evaluate CA-CBT in more diverse clinical populations, including individuals with lower digital literacy or greater medical complexity.

Additional limitations include the unblinded design, which may have introduced performance or reporting bias despite standardized procedures, and the relatively small sample size, which limits statistical power and precision of estimates. Sample size assumptions were informed by effect estimates from a conceptually related disease-specific outcome, AFEQT, rather than prior SAQ-based CBT trials in post-MI populations, as such data were limited at the time of study design. Furthermore, although multiple SAQ subscales were specified as primary end points to capture different dimensions of disease-specific health status, the power calculation did not take the use of 4 outcomes into account; findings should therefore be interpreted with consideration of these constraints in mind. Finally, the 12-month follow-up reflects within-group observations rather than randomized long-term effects.

## Conclusions

This randomized trial provides initial evidence that a targeted, digitally delivered, exposure-based CBT intervention can improve disease-specific health status and reduce cardiac anxiety in patients with prior MI. The intervention was delivered safely with high adherence and participant satisfaction, supporting feasibility in a post-MI population. These findings highlight the potential value of targeting cardiac-specific fear and avoidance through systematic exposure within a multidisciplinary framework. Future studies should include active comparators, assess longer-term outcomes, and evaluate strategies for implementation in routine post-MI care.

## Funding support and author disclosures

This study was supported by the Swedish Heart and Lung Foundation (Nr: 20210779) and the 10.13039/501100004359Swedish Research Council (Nr: 2022-00895). The funding body had no involvement in the design of the study, data analysis, or interpretation of the results. Dr Braunschweig has received fees for trial committee participation and lectures by Biotronik, Biosense Webster, Abbott, Novartis, Astra Zeneca, Bayer, Boehringer, and Pfizer, all of which were directly payed to his employer. Dr Ljótsson has co-authored a Swedish self-help book on exposure-based cognitive behavior therapy for health anxiety and has a publishing agreement with Cambridge University Press for a self-help book for irritable bowel syndrome. Dr Mellbin has received lectures, consulting, and clinical trials fees to her institution from Amarin, Amgen, Astra Zeneca, Bayer AG, Boehringer-Ingelheim Janssen, Novartis, NovoNordisk, and Sanofi. All other authors have reported that they have no relationships relevant to the contents of this paper to disclose.
