# Supplementary material for: Digital Cognitive Behavioral Therapy for Cardiac Anxiety After Myocardial Infarction: Effects on Disease-Specific Health Status–Detailed Methods and Protocol
Source: JACC Adv. 2026 Mar 25;5(4):102669. doi: 10.1016/j.jacadv.2026.102669 (PMC13131430; doi:10.1016/j.jacadv.2026.102669)
Supplement: Supplemental Material [file mmc1.docx]

**Supplementary Material**

[Supplementary Methods 3](#_Toc221190231)

[Recruitment and determination of eligibility 3](#_Toc221190232)

[Recruitment materials 3](#_Toc221190233)

[Study nurse eligibility and clinical assessment (Selected excerpts) 5](#_Toc221190235)

[Baseline clinical characteristics (Selected variables) 6](#_Toc221190236)

[Study psychologist eligibility and clinical assessment 6](#_Toc221190237)

[Study cardiologist eligibility review 9](#_Toc221190238)

[Differential diagnostic considerations 9](#_Toc221190239)

[Treatment content 10](#_Toc221190240)

[Supplemental Textbox 1 Treatment content for each module 10](#_Toc221190241)

[Excerpts from psychoeducation on cardiac anxiety 11](#_Toc221190242)

[Participant safety and multidisciplinary collaboration 14](#_Toc221190243)

[Participant education on cardiac sensations 14](#_Toc221190244)

[Excerpt of instructions participant safety to study psychologist during treatment 15](#_Toc221190245)

[Clinical consultation and supervision procedures 16](#_Toc221190246)

[Exluded participants 16](#_Toc221190247)

[Supplemental Table 1. Demographic characteristics of excluded applicants 16](#_Toc221190248)

[Examples of inclusion and exclusion decisions 17](#_Toc221190249)

[Secondary outcome measures 19](#_Toc221190250)

[Self-reported outcomes 19](#_Toc221190251)

[Physical activity behaviors measured by accelerometry 20](#_Toc221190252)

[Cardiac-related biomarkers 20](#_Toc221190253)

[Statistical analysis 21](#_Toc221190254)

[Software 21](#_Toc221190255)

[SAQ analysis 21](#_Toc221190256)

[Cardiac healthcare visits 22](#_Toc221190257)

[Accelerometer analysis 22](#_Toc221190258)

[Twelve-month follow-up analysis 22](#_Toc221190259)

[Sensitivity analysis 22](#_Toc221190260)

[Supplementary Results 24](#_Toc221190261)

[Supplemental Table 2. Clinical outcome measures 24](#_Toc221190262)

[Supplemental Table 3. Estimated between-group differences in mean change 25](#_Toc221190263)

[Supplemental Table 4. Estimated between-group differences in mean change 26](#_Toc221190264)

[Supplemental Table 5. Estimated within-group differences in mean change 27](#_Toc221190265)

[Supplemental Figure 1. Waterfall Plots 29](#_Toc221190266)

[Supplemental Table 6. Cohen’s d effect sizes 31](#_Toc221190267)

[Cardiac specific health-care utilization 33](#_Toc221190268)

[Usual care context – health-care visits 34](#_Toc221190269)

[Psychologist/psychotherapist visits 34](#_Toc221190270)

[Change in psychotropic medication 35](#_Toc221190271)

[Changes in cardiac health 35](#_Toc221190272)

[References 36](#_Toc221190273)

# ****Supplementary Methods****

## **Recruitment and determination of eligibility**

## **Recruitment materials**

**Flyer:

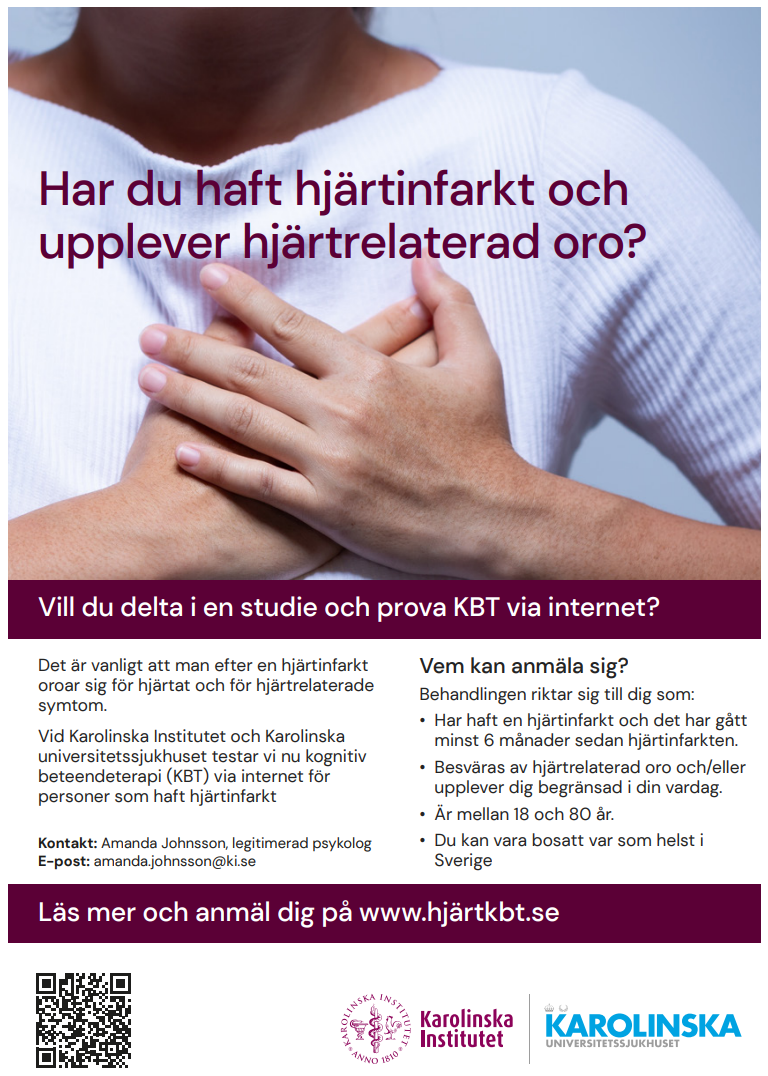
**

## **Translation of flyer:**

Have you had a myocardial infarction and experience heart‑related worry?
It is common to feel concerned about the heart and heart‑related symptoms after a myocardial infarction. At Karolinska Institutet and Karolinska University Hospital, we are currently evaluating internet‑delivered cognitive behavioral therapy (CBT) for individuals who have had a heart attack.

Would you like to participate in a study and try CBT delivered online?
Read more and register at: www.hjartkbt.se

Who can participate?
This treatment is intended for individuals who:
• Have had a myocardial infarction, with at least 6 months having passed since the event
• Experience heart‑related worry and/or feel limited in their daily life
• Are between 18 and 80 years old
• Live anywhere in Sweden

Contact:
Amanda Johnsson, Licensed Psychologist
Email: amanda.johnsson@ki.se

*Digital advertising newspaper*

*
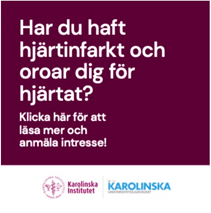
*

Translation of digital advertising newspaper:

Have you had a heart attack and feeling worry about your heart? Click here to read more and to sign up!

Advertisement social media

Translated advertisement text example:

It is common to experience heart‑related worry after a heart attack. Researchers at Karolinska Institutet are studying how internet‑delivered cognitive behavioral therapy (CBT) may improve quality of life and reduce heart‑related worry in individuals who have had a heart attack. Are you interested in learning more? Read more and register for the study here:

## **Study nurse eligibility and clinical assessment (Selected excerpts)**

The study nurse conducted a structured telephone-based eligibility and clinical assessment to confirm medical stability prior to inclusion. The assessment included the following components: **- Core eligibility criteria:** age 18-80 years; type 1 myocardial infarction ≥6 months prior to enrollment; consent for study team access to medical records (physician, psychologist, study nurse). **- Myocardial infarction history:** symptom presentation during the myocardial infarction (chest pain-predominant vs non-chest pain-predominant) and whether the patient recognized the event at the time. **- Recent or planned medical changes:** medication changes within the past month; recent cardiac procedures (e.g., angiography/PCI, CABG); and planned procedures or medication changes within the subsequent 3 months.
**- Relevant conditions:** medical conditions limiting physical activity or requiring avoidance of exertion; alcohol dependence reasonably excluded based on screening; **current use of psychotropic medication (e.g., selective serotonin reuptake inhibitors), with required dose stability for at least 1 month prior to enrollment and no planned changes during the treatment period.
- Medical record review:** availability of recent ECG, echocardiogram, and blood pressure measurement (≤3 months).

## **Baseline clinical characteristics (Selected variables)**

- **Anthropometrics:** height, weight, body mass index
- **Vital signs:** systolic and diastolic blood pressure, resting heart rate
- **Cardiac status:** myocardial infarction type (STEMI/NSTEMI), left ventricular ejection fraction (>35% vs ≤35%), presence of significant valvular disease
- **Laboratory values:** HbA1c, LDL cholesterol
- **Symptoms:** dyspnea at rest and during exertion
- **Comorbidities:** hypertension, diabetes, atrial fibrillation, heart failure, angina, prior stroke or TIA, sleep apnea, COPD, thyroid disease
- **Prior cardiac history:** previous myocardial infarction, revascularization (PCI/CABG), ablation, pacemaker
  - **Medications:** antiplatelet therapy (ASA, DAPT), beta-blockers, ACE inhibitors/ARBs, lipid-lowering therapy, anticoagulants, and psychotropic medication (e.g., SSRIs)

## Study psychologist eligibility and clinical assessment (Representative questions)

Clinical psychologists conducted a structured telephone-based clinical assessment to confirm the presence of clinically meaningful cardiac anxiety and to evaluate psychiatric comorbidity relevant to study eligibility. The assessment focused on differentiating cardiac anxiety from other anxiety or mood disorders and on identifying exclusionary conditions requiring specialized care. Psychologists were instructed to review self-reported screening data and the study nurse’s medical assessment as part of this evaluation.
Assessment of cardiac anxiety
**- Could you tell me about when you had your heart attack and what that experience was like for you?**

**- What has been most difficult for you since the heart attack?** Do you feel that the heart attack, or worry or anxiety about your heart, affects your life?

**- When you notice heart-related symptoms or sensations** (for example, chest pain, shortness of breath, or a rapid heartbeat), **how do you usually respond?** (Examples: resting, stopping activities, checking your pulse, seeking medical care, withdrawing, feeling irritated or hopeless.)

**- In what ways has the heart attack affected your daily life?** Are there activities you have stopped or avoided because of worry about your heart or heart-related symptoms? (Examples: avoiding physical activity, travel, strong emotions, alcohol, lying on the left side, or being alone at home.)

**- What do you usually think when you notice heart-related symptoms or sensations?** Do you have recurring worries about your heart health? If so, could you describe some of them? (Examples: fear of medical consequences, not being able to cope, dying, or having another heart attack.)

**- Do you do anything to avoid activating your heart or to monitor it?** (Examples: avoiding stress, resting, monitoring physical activity, following strict routines for sleep or diet, breathing calmly.)
Question for assessor: Does the patient experience a disproportionately high and persistent level of anxiety regarding their cardiac health or symptoms? Does the patient report any avoidance behavior or impairment in daily life due to cardiac anxiety?

Differential diagnostic considerations

Review each symptom domain and assess whether symptoms are present that contribute to clinically meaningful distress or functional impairment. Note: A structured diagnostic interview should be used when further assessment is indicated or when an exclusionary psychiatric condition is suspected.

- **Depressive symptoms** (e.g., persistent low mood, loss of interest or pleasure)
  - In the past month, have you experienced thoughts of not wanting to live?
  - Assess current and past suicidal ideation.
- **Excessive worry / generalized anxiety** (e.g., frequent or uncontrollable worry, worry about minor matters)
- **Social anxiety** (e.g., distress or avoidance in social situations)
- **Panic symptoms** (e.g., sudden episodes of intense anxiety or discomfort)
- **Agoraphobic avoidance** (e.g., avoidance of places or situations due to fear of panic attacks or heart-related symptoms)
- **Obsessive–compulsive symptoms** (e.g., recurrent intrusive thoughts, urges, or repetitive behaviors)
- **Sleep problems** (e.g., persistent sleep disturbance with impact on daily functioning)
- **Stress-related symptoms**
- **Trauma-related symptoms** (e.g., exposure to severe or life-threatening events, re-experiencing, avoidance, or hyperarousal)
- **Alcohol use** (review AUDIT scores)
- **Other psychiatric or psychological conditions**, if present

Question for the assessor: Do these psychological symptoms reflect cardiac anxiety or a separate condition requiring targeted treatment?

## §Study cardiologist eligibility review

Checklist (all must be YES for the patient to be eligible for CBT treatment)

| **Criteria** | **Yes** | **No** |
| --- | --- | --- |
| Myocardial infarction > 6 months ago |  |  |
| Able to be physically active (Any other condition that prevents or should prevent physical exertion, which is part of the treatment—such as climbing a few stairs, jogging in place—must be reasonably ruled out, as well as significant heart failure.) |  |  |
| On medication according to guidelines |  |  |
| Current ECG within inclusion criteria |  |  |
| No clinical suspicion of heart failure (EF < 35%) or significant valvular disease |  |  |
| Blood pressure within inclusion criteria |  |  |

Decision

I hereby certify that, based on the above, the patient can participate in CBT treatment.

☐ The patient CAN participate in CBT treatment

☐ The patient CANNOT participate in CBT treatment

## Differential diagnostic considerations

Cardiac anxiety, that is excessive fear of recurrent cardiac events, fear and hypervigilance towards cardiac-related sensations, and related avoidance behavior ^1^, is conceptually distinct from anxiety disorders as defined in DSM-5^2^. Accordingly, the psychological assessment incorporated differential diagnostic considerations, described below, to distinguish cardiac anxiety from related psychiatric conditions. In contrast to cardiac anxiety, generalized anxiety disorder involves diffuse worry themes across multiple life domains, whereas panic disorder centers on fear of panic attacks and their anticipated consequences. Health anxiety reflects a persistent fear of serious illness that may include, but is not limited, to cardiac concerns. Cardiac-induced post-traumatic stress disorder (PTSD) ^3,4^ and cardiac anxiety may co-occur, but cardiac-induced PTSD is more anchored to the traumatic experience of the acute cardiac episode and its re-experiencing.

These distinctions underscore that cardiac anxiety is conceptually and clinically distinct from other anxiety and psychiatric disorders, and differential diagnostic considerations were therefor incorporated into the psychological eligibility assessment.

## Treatment content

### Supplemental Textbox 1 Treatment content for each module

| Module 1 – Introduction and psychoeducation | - Psychoeducation on psychological responses after myocardial infarction - How cardiac anxiety and avoidance behaviors hinders recovery and are reinforced - Goal setting and initial mapping of avoidance, control and safety behaviors - Behavioral analysis |
| --- | --- |
| Module 2 – Interoceptive exposure | - Introduction to and rationale for exposure - Interoceptive exercises to safely evoke cardiac-related sensations and reduce associated fear - Introduction to labeling of bodily sensations, emotions and thoughts - Continous behavioral tracking |
| Module 3 – Situational exposure | - Introduction to situational exposure (in-vivo) - Gradual exposure to avoided situations, places, and activities - Strategies to reduce avoidance, control and safety behaviors (response prevention) - Continued work with labeling and behavioral tracking |
| Module 4 – Cardiac anxiety and worry thoughts | - Understanding and addressing cardiac-related worry - Application of labeling exercise in managing thoughts and worry - Continued interoceptive and situational exposure, response prevention, behavioural tracking and labeling |
| Modules 5, 6, 7 – Continuing exposure and reclaiming activities | - Continued exposure, response prevention and labeling - Combining interoceptive and situational exposure |
| Module 8 – Summary and relapse prevention | - Review of progress and treatment strategies - Identifying high-risk situations - Developing a personalized plan for maintaining gains |

## **Excerpts from psychoeducation on cardiac anxiety**

The following text provides illustrative excerpts from the patient-facing treatment to demonstrate the psychoeducational content on cardiac anxiety as well as examples of common avoidance behaviors.

**Fear of heart-related symptoms**

After a heart attack, it is common to worry about bodily sensations and stress-related symptoms, such as pressure or discomfort in the chest, shortness of breath, chest pain, palpitations, or pain in the upper back or between the shoulder blades. The symptoms themselves are often harmless, but they may trigger fear of having another heart attack and lead to avoiding physical activity. They can also remind you of the symptoms you experienced during the heart attack itself. Being constantly alert to bodily sensations and managing worry about another heart attack can be exhausting and may make everyday life feel limited and restricted. Many people also feel uncertain about how much they can safely strain their heart and body after a heart attack. You may have received information about the importance of lifestyle changes and physical activity, while at the same time feeling unsure about how to exercise safely or how much exertion is appropriate. This uncertainty can lead to a loss of confidence in your body and in your heart. **Heart-related avoidance behaviors**

Many people develop avoidance and control behaviors in an attempt to prevent heart-related symptoms or to feel more in control of their heart. Some describe constantly checking how their heart feels and worrying about whether it is functioning properly. Others notice that they focus on their heart more than they want to and become highly alert to heart-related signals.

Over time, this often leads to feeling increasingly controlled and limited by heart-related worry. The heart works continuously—every minute, day and night—and there is natural variation in how fast and forcefully it beats, as well as in how much we notice it. It is therefore unavoidable to sometimes feel extra beats or strong palpitations, which are usually harmless. Worry and stress can also naturally make the heart beat faster, which may further increase anxiety. This can lead to understandable but unhelpful cycles that gradually restrict daily life. When heart-related sensations occur, many people feel prepared to stop what they are doing, becoming highly focused on their body. This can make it difficult to concentrate and to fully engage in activities that are meaningful and important. **Common things people do after a heart attack to reduce worry or control their heart:**

 Checking your pulse often (by hand or with a smartwatch)

 Avoiding activities that make your heart beat faster

 Avoiding exercise or physical activity

 Avoiding running, even for short things like catching a bus

 Avoiding strong emotions

 Avoiding sexual activity

 Avoiding social events or being around people

 Avoiding places far from a hospital or clinic

 Avoiding lying on your left side

 Traveling less or stopping trips

 Slowing down or stopping when you notice heart, chest, or back sensations

 Worrying about medical problems or complications

 Avoiding being alone at home or walking alone

 Worrying that something is seriously wrong with your heart

 Resting before and after activities more than usual

 Saying no to invitations or social activities

 Avoiding planning activities because you’re unsure how you’ll feel

 Avoiding thinking about the future or enjoyable plans

 Thinking a lot about death or missing out on future life events

 Avoiding new challenges or physical activities out of fear

 Cutting down on commitments to feel safer or less stressed

**Mats, 58 years old, describes his experience after a heart attack**

“I had a difficult spring, both at work and in my personal life. One day at work, I suddenly developed severe chest pain. I went to the primary care clinic, and they quickly sent me by ambulance to the hospital. Once I arrived, everything happened very fast. For a few days, I was closely monitored and underwent many tests. It still feels unreal that this happened to me. I feel frustrated that it occurred, and the worry has stayed with me. I often feel pressure in my chest and become afraid that something acute might happen to my heart again. My cardiologist says everything looks normal, but I still find it hard to let go of the worry. I go for walks, but I often stop, check how I feel, and try to calm my heart. I often feel guilty that I should exercise more and deal with things better, but my energy just isn’t there. I want to let go of the worry and feel like my old self again.”

**Understanding the pattern**

Like Mats, many people respond to heart-related sensations with worry and avoidance. After a heart attack, it is common to become highly attentive to bodily signals and to interpret them as dangerous, even when they often have a natural and harmless explanation. Avoidance can gradually make life feel more restricted and prevents opportunities to learn that these sensations can be managed safely. This treatment aims to help you break this pattern and regain confidence in your body and your heart

## Participant safety and multidisciplinary collaboration

## Participant education on cardiac sensations

Patients received psychoeducation distinguishing benign cardiac-related sensations from symptoms warranting medical evaluation. Examples of commonly discussed benign sensations included extra beats or palpitations occurring during stress or caffeine intake, transient sensations of a “pause” followed by a stronger heartbeat reflecting normal cardiac filling, and dizziness or chest discomfort related to anxiety-driven hyperventilation or musculoskeletal tension. These examples were used to reduce misinterpretation of bodily sensations and support safe engagement in exposure exercises. Patients were provided with clear instructions regarding symptom escalation and when to seek medical attention to ensure safety.

*Excerpt from instruction on when to seek medical care*

When Should You Seek Medical Care?

Consult your treating physician promptly if:

- You experience new-onset or exertion-induced chest pain. Dizziness presenting as near-fainting without warning may sometimes be due to a low heart rate.
- Chest pain worsens (including nocturnal pain), even if it resolves after taking nitroglycerin.

Seek immediate medical attention if you:

- Have a pressing or cramping chest pain lasting longer than 15 minutes and cannot identify the cause.
- Experience chest pain along with shortness of breath and cold sweats, or chest pain combined with irregular heartbeats.
- Have chest pain and have taken nitroglycerin three times at 5-minute intervals without relief. In this case, seek emergency care immediately and call emergency services (e.g., 112).

## Excerpt of instructions regarding participant safety to study psychologist during treatment

**Points to consider during psychological treatment delivery**

- **Comorbid medical conditions:** Patients frequently present with somatic comorbidities. Exposure exercises should therefore be planned and adapted according to individual physical capacity.
- **Sensitivity during exposure:** Particular clinical attention is required during exposure exercises, especially during interoceptive exposure (Module 2), to ensure patient safety and appropriate pacing.
- **Angina symptoms:** Psychologists should remain alert to patient reports suggestive of angina during treatment.
- **Monitoring of new or persistent symptoms:** New-onset or persistent symptoms warrant careful evaluation. For example, chest pain lasting ≥15 minutes should prompt immediate clinical consideration.
- **Nitroglycerin use:** If nitroglycerin prescribed for angina is available, patients are generally advised to administer one dose and monitor symptom response. If symptoms persist or recur after several minutes, a second dose may be taken. If symptoms do not resolve, emergency medical services should be contacted (eg, 112). This guidance reflects standard clinical recommendations and is not study-specific.
- **Symptoms requiring caution and escalation:** Participants receive guidance regarding symptom monitoring and escalation in the treatment manual (Module 1).
- For **new-onset or exertion-related chest pain**, even if brief, consultation with the study team is recommended. **Dizziness associated with near-syncope or syncope**, particularly if occurring without warning, may indicate clinically relevant bradycardia and should prompt consultation with the study team. The symptoms described above warrant **temporary discontinuation of exposure exercises** until medical consultation is complete.

## **Clinical consultation and supervision procedures**

Participant safety was prioritized throughout treatment delivery, and all procedures were conducted in accordance with the study protocol. Psychologists were instructed to contact the lead psychologists (A.J., J.S.) and the study nurse (E.Ó.) with any questions regarding treatment progression or patient-reported symptoms. Clinical questions were communicated via the platform’s supervision messaging function, with notification by email when appropriate. The study nurse reviewed incoming queries and involved the study cardiologist as needed. Responses were provided within the supervision messaging system.

When uncertainty arose regarding symptom interpretation or exposure progression, psychologists coordinated with the lead psychologists and study nurse, who consulted the study cardiologist before further exposure was implemented.

## Exluded participants

### **Supplemental Table 1.** Demographic characteristics of excluded applicants and included participants

|  | Excluded  (n=76) | Included  (n=96) |
| --- | --- | --- |
| Age - yr | 66±9.8 | 63.6±9.3 |
| Gender |  |  |
| Female | 30 (39%) | 26 (27%) |
| Male | 46 (61%) | 70 (73%) |
| Employment status |  |  |
| Employed | 22 (29%) | 56 (58%) |
| Retired | 47 (62%) | 43 (45%) |
| Unemployed | 2 (3%) | 2 (2%) |
| Sick leave | 5 (7%) | 6 (6%) |
| Student | 0 (0%) | 2 (2%) |
| Highest completed education |  |  |
| Less than high school | 5 (7%) | 5 (5%) |
| High school | 15 (20%) | 19 (20%) |
| Post high school, not university | 18 (24%) | 17 (18%) |
| University | 38 (50%) | 55 (57%) |

**Note:** Values are mean ± SD or n (%) **Abbreviations:** Yr: Year

## Examples of inclusion and exclusion decisions

Example 1 – Exclusion (Planned medical procedure)
Age/Sex: Male, 67 years
Summary: Had myocardial infarction 8 months ago. Reports cardiac anxiety but is scheduled for CABG surgery within the next 2 months. Planned medication changes and follow-up visits confirmed.
Decision: Excluded – Upcoming major intervention prevents participation.
Action: Patient informed; advised to return after recovery if interested.

Example 2 – Inclusion (Stable medical status)
Age/Sex: Female, 58 years
Summary: MI occurred 12 months ago. No planned procedures or medication changes. EF >35%, stable blood pressure and heart rate. No severe comorbidities limiting physical activity.
Decision: Included – Meets all medical criteria for participation.
Action: Patient informed; scheduled for psychological assessment.

Example 3 – Exclusion (Alcohol dependence)
Age/Sex: Female, 64 years
Summary: MI occurred 7 years ago. AUDIT score (>20 points) and psychologist interview indicates alcohol dependence. Cardiac anxiety with avoidance of places far from hospital, fear of being alone and avoidance of physical activity. Requires addiction treatment before participation.
Decision: Excluded – Safety concern; needs alternative intervention.
Action: Patient informed; advised to seek support via primary care or addiction services.

Example 4 – Exclusion (No cardiac anxiety)
Age/Sex: Male, 60 years
Summary: MI occurred 2 years ago. Reports feeling well after myocardial infarction. Reports no increased worry about the heart, cardiac anxiety or avoidance behaviors. Uses apple watch to monitor pulse out of interest, not due to worry. Denies persistent worry thoughts or functional impairment.
Decision: Excluded – Does not meet criteria for intervention.
Action: Patient informed; advised to continue routine care if needed.

Example 5 – Inclusion (Cardiac anxiety and avoidance)
Age/Sex: Female, 55 years
Summary: MI occurred 5 years ago. Reports frequent worry about heart health and fear of another infarction. Avoids physical activity and traveling due to anxiety. Describes recurrent thoughts and fear of dying and monitors symptoms daily. Also monitors exact location to alert ambulance if necessary. Clinical levels of cardiac anxiety
Decision: Included – Suitable for CBT intervention.
Action: Patient informed; scheduled for pre-measurements.

Example 6 – Exclusion (Severe depression)
Age/Sex: Male, 62 years
Summary: MI occurred 7 months ago. Reports persistent low mood, loss of interest, and suicidal thoughts recurrent daily in past month. PHQ-9 score: 23 (severe depression). Requires psychiatric care before participation.
Decision: Excluded – Safety concern; needs alternative intervention.
Action: Patient informed; advised to contact primary care for psychiatric referral.

## Secondary outcome measures

## Self-reported outcomes

Self-reported questionnaires were administered online at baseline, weekly during the eight-week intervention period, post-treatment and at three-month follow-up for all participants. An additional 12-month follow-up assessment was conducted for the CA-CBT group. The following questionnaires were administrated: The Cardiac Anxiety Questionnaire (CAQ)^5^ measures cardiac anxiety through 18 questions, each rated on a five-point scale and assesses three dimensions of cardiac anxiety: fear/worry, avoidance, and attention. Higher scores indicate greater levels of cardiac anxiety, both for the individual dimensions and the overall scale. Both the subscales and the full scale have shown strong validity in a Swedish post-MI population. ^6^. The Perceived Stress Scale-4 (PSS-4)^7^ measures stress levels through four questions that evaluate the frequency of stress-related symptoms. Higher summary scores indicate greater perceived stress. To measure cardiac-related symptoms not captured elsewhere participants also reported symptoms in four items from Symptom checklist Severity and Frequency Scale (SCL)^8^ and University of Toronto Atrial fibrillation Severity Scale (AFSS) ^9^. The Body Sensation Questionnaire (BSQ)^10^ assesses fear of common anxiety-related symptoms (e.g., palpitations, dizziness, numbness) through 17 items, each rated on a five-point scale based on the level of fear associated with the symptom. The Godin Leisure-Time Exercise Questionnaire (GLTEQ)^11^, is used to assess physical activity levels. Participants report the frequency and intensity of their physical activities, which are then categorized as either "active" or "insufficiently active”. To asses depressive symptoms, we used Patient Health Questionnaire-9 (PHQ-9)^12^. This tool consists of nine questions that evaluate the frequency of common depressive symptoms and their impact on daily life. The Generalized Anxiety disorder-7 (GAD-7)^13^ measures common symptoms of worry and generalized anxiety in seven items. The 12 Short-Form Health Survey (SF-12) ^14^ measures health-related QoL, and are summarized in a Mental Health Summary score (SF-12 MCS) and a Physical Health Summary score (SF-12 PCS) between 0 to 100.^14^ To describe general health‑care utilization, we used the TIC‑P A‑section, which captures the type and frequency of contacts with health‑care providers (e.g., physicians, nurses, physiotherapists) during the preceding 4‑week period.^15^ To evaluate health-care seeking, we used three items on cardiac-specific healthcare consumption during the preceding three months: ER-visits, hospitalization due to cardiac-related problems and cardiologist visits from University of Toronto Atrial fibrillation Severity Scale; (AFSS).^9^ The study also included other measures (e.g. health-economic data collection, measurement of pain-sensitivity), that will be reported elsewhere.

## Physical activity behaviors measured by accelerometry

**At baseline, post-treatment and three-month follow-up participants were asked to wear an accelerometer (ActiGraph® - model wGT3X-BT) on their non-dominant wrist for seven consecutive 24hr-periods**. The accelerometer was programmed to register accelerations at 90 Hz (epoch length 10 seconds), and participants were instructed to wear the accelerometer at all times, except when showering or bathing. Participants who visited the Karolinska University Hospital received the accelerometer during their visit, while those unable to attend the clinic received it by mail. All participants returned the accelerometer by mail. The raw data was then downloaded using the ActiLife software (ActiGraph LCC) and processed with the open-source GGIR R package ^16^ as previously described ^17^. Raw accelerations were aggregated as the Euclidean Norm of the raw accelerations with negative values rounded to zero in 5-s epochs. Appropriate thresholds were used to identify physical activity intensities (e.g., ≥100 mg for MVPA) ^18^.

## Cardiac-related biomarkers

Venous blood samples were collected at baseline, post-treatment, and at the 3-month follow-up. All patients were systematically invited to provide a blood sample, the final sample included those able to attend the clinic. At each time point, 20 ml of venous blood were collected by the cardiac study nurse (E.Ó.) and processed according to standardized procedures. Participants fasted from midnight to the time of the visit, scheduled during the morning of the following day. Samples were collected in EDTA tubes (HbA1c) and Li-heparin tubes (TC, LDL, HDL, triglycerides, hsCRP, creatinine). HbA1c samples were processed immediately, while all other samples were stored at –80 °C in the Cardiac Research Unit, part of Karolinska Institutet’s biobank, until completion of the 3-month follow-up.

## Statistical analysis

## Software

Analyses were performed in R, using the libraries nlme (for linear mixed models analysis), lme4 (for generalized linear mixed models analysis), metafor (for calculation of effect sizes), and emmeans (for extraction of group contrasts from linear mixed models and ANCOVAs) by author B.L. Analytic code (R scripts) is available upon reasonable request.

## SAQ analysis

According to the scoring instructions for SAQ, the angina stability subscale, which measures improvement in frequency of angina symptoms, should be scored as missing if participants have not had any angina symptoms during the past four weeks. However, this led to a large number of missing values at the follow-up measurements, potentially because symptoms had decreased over time. Therefore, we adjusted the scoring and participants who indicated no angina symptoms for the last for weeks were assigned a stability score of 100, indicating maximum improvement. One participant provided an outlying very low score, 2.8, on the SAQ physical limitation subscale at the post-treatment assessment. Including this score of 2.8 on the physical limitation subscale at *post-treatment*, led to a 96% increase in residual (unexplained) variance of the linear mixed model and a non-significant between-group effect, p=.057, at *three-month follow-up*, whereas replacing this score with a missing value led to a significant between-group effect, p=.008, at three-month follow-up. Importantly, this individual had scored 100 (no physical limitation) at baseline, the four weekly SAQ ratings preceding the post-treatment assessment, and at three-month follow-up. We judge that the 2.8 score was most probably due to misinterpretation of the direction of the scale and this post-treatment score was therefore removed and regarded a missing value.

## Cardiac healthcare visits

The cardiac healthcare visits outcome did not follow a normal distribution and had a large number of zero visits. We therefore employed generalized linear mixed models using a Poisson distribution and a log link function, in the analysis of cardiac health care visits. The cardiac healthcare visits model included the same independent variables as the primary analysis (group, time, and time*group interaction effect) and also included a random intercept.

## Accelerometer analysis

Participants were included in the accelerometer analysis if they had at least one valid day of accelerometer data, defined ^16^. To account for variations in activity levels across weekdays and weekends, we calculated the average Moderate to Vigorous Physical activity (MVPA) using a weighted mean of both.

## Twelve-month follow-up analysis

To evaluate the 12-month follow-up, we examined within-group changes in the CA-CBT group. In this analysis we used mixed models, with time as a four-level factor consisting of baseline (reference value), post-treatment, and 3-month and 12-month follow-ups. Analyses at 12-month follow-up were restricted to the CA-CBT group.

## Sensitivity analysis

In response to reviewers’ request for sensitivity analyses, we performed a set of ANCOVA analyses where we controlled for baseline values of the outcome of interest and investigated if baseline characteristics moderated the treatment effects. These analyses were performed on the SAQ QoL and physical limitation subscales and the CAQ at 3-month follow-up (primary endpoint). We performed two sets of analyses. First, we performed ANCOVAs with only the baseline value of the outcome and group as independent variables. In the second set of sensitivity analyses, we included baseline characteristics that could potentially moderate treatment effects. In these analyses, we included the baseline value, group, and group*moderator as independent variables. The candidate moderators were age, number of years since last MI, employment status, CAQ baseline score (not included as a moderator in CAQ analysis). For the employment status, we collapsed the groups into employed (including employed and full-time students) and unemployed (including retirees and unemployed). All moderators were mean-centered before analysis.

## **Supplementary Results**

### **Supplemental Table 2.** Clinical outcome measures: Observed means and standard deviations, estimated effects sizes.

|  | Raw score at baseline | | Raw score at post | | Raw score at 3MFU | | Least square mean change from baseline to 3MFU | | Est diff in mean change baseline to 3MFU | P-value | |
| --- | --- | --- | --- | --- | --- | --- | --- | --- | --- | --- | --- |
|  | CA-CBT | UC | CA-CBT | UC | CA-CBT | UC |  |  |  |  |  |
| **Outcome** | (n=48) | (n=48) | (n=45) ^a^ | (n=47) | (n=45) ^a^ | (n=46) | CA-CBT | UC |  | Raw | FDR |
| SCL frequency | 16.7 ± 8.4 | 20.0 ± 8.8 | 13.0 ± 7.5 | 17.1 ± 7.8 | 12.7 ± 8.1 | 16.5 ± 8.6 | -3.9 ± 0.7 | -3.5 ± 0.7 | -0.4 (-2.5 to 1.6) | .667 | .757 |
| SCL severity | 14.8 ± 7.0 | 18.1 ± 8.1 | 10.8 ± 6.4 | 14.9 ± 7.0 | 11.1 ± 7.1 | 14.3 ± 7.2 | -3.6 ± 0.7 | -3.9 ± 0.7 | 0.2 (-1.7 to 2.1) | .816 | .816 |
| SF12 Mental | 43.1 ± 13.1 | 40.6 ± 10.2 | 48.7 ± 11.3 | 43.7 ± 10.0 | 49.0 ± 11.0 | 43.1 ± 11.3 | 5.8 ± 1.4 | 2.7 ± 1.4 | 3.2 (-0.9 to 7.1) | .122 | .262 |
| SF12 Physical | 47.5 ± 8.5 | 46.2 ± 8.2 | 50.4 ± 9.1 | 46.7 ± 9.0 | 49.4 ± 9.3 | 47.6 ± 7.7 | 1.89 ± 1.0 | 1.3 ± 1.0 | 0.5 (-2.2 to 3.3) | .707 | .757 |
| PSS-4 | 6.3 ± 3.0 | 6.7 ± 3.2 | 4.6 ± 3.1 | 5.4 ± 3.2 | 4.9 ± 3.3 | 5.8 ± 3.1 | -1.5 ± 0.4 | -0.8 ± 0.4 | -0.7 (-1.8 to 0.5) | .245 | .460 |

**Note:** Raw scores are presented as mean±SD. Changes in least-square means from baseline to the three-month follow-up (primary endpoint) and the estimated difference at three months were derived from linear regression mixed models. The least-square mean changes are shown as estimated change ± SE. The estimated differences are presented with 95% confidence intervals. **Abbrevations:** 3MFU: Three-month follow-up, CA-CBT: CBT for Cardiac Anxiety, UC: Usual Care, FDR: P-value corrected for the False Discovery Rate, SCL: Symptom Checklist, SF-12 PCS: Short-Form Health Survey SF-12 Physical Health Summary score, SF-12 MCS: Short-Form Health Survey Mental Health Summary score, PSS-4: Perceived Stress Scale-4. ^a^ (n=45) One participant provided partial data due to perceived assessment burden.

### **Supplemental Table 3.** Estimated between-group differences in mean change at post-treatment and at three-months follow-up.

|  | Raw score at baseline | | Raw score at post | | Raw score at 3MFU | | Least square mean change from baseline to 3MFU | | Est diff in mean change baseline to 3MFU | P-value | |  |
| --- | --- | --- | --- | --- | --- | --- | --- | --- | --- | --- | --- | --- |
|  | CA-CBT | UC | CA-CBT | UC | CA-CBT | UC |  |  |  |  |  |  |
| **Outcome** | (n=46) | (n=48) | (n=39) | (n=41) | (n=37) | (n=44) | CA-CBT | UC |  | Raw | FDR | d |
| Moderate to vigorous physical activity | 35.9 ± 27.6 | 43.4 ± 35.0 | 35.9 ± 30.4 | 38.9 ± 27.6 | 33.9 ± 23.0 | 38.2 ± 31.1 | -4.7 ± 3.0 | -4.4 ± 2.8 | -0.3 (-8.4 to 7.7) | .934 | .934 | 0.01 |
| Light physical activity | 157.2± 45.6 | 156.0± 41.4 | 163.0 ± 41.8 | 161.7 ± 41.2 | 157.5 ± 45.5 | 159.5 ± 38.9 | 2.6 ± 5.3 | 4.9 ± 4.9 | -2.3 (-16.6 to 12.0) | .751 | .934 | 0.05 |
| Sedentary time | 777.5 ± 111.1 | 743.9 ± 85.3 | 760.0 ± 107.7 | 767.5 ± 79.7 | 757.2± 81.9 | 745.5 ± 89.8 | -20.3 ± 10.3 | -4.5 ± 9.6 | -15.7 (-43.6 to 12.13) | .267 | .934 | 0.16 |

**Note:** Raw scores are presented as mean±SD. Changes in least-square means from baseline to the three-month follow-up (primary endpoint) and the estimated difference at three months were derived from linear regression mixed models. The least-square mean changes are shown as estimated change ± SE. The estimated differences are presented with 95% confidence intervals. Effect sizes are shown as d, calculated as Cohen’s d. **Abbrevations:** 3MFU: Three-month follow-up, CA-CBT: CBT for Cardiac Anxiety, UC: Usual Care, FDR: P-value corrected for the False Discovery Rate.

### **Supplemental Table 4.** Estimated between-group differences in mean change at post-treatment and at three-months follow-up.

|  | Raw score at baseline | | Raw score at post | | Raw score at 3MFU | | Least square mean change from baseline to 3MFU | | Est diff in mean change baseline to 3MFU | P-value | |  |
| --- | --- | --- | --- | --- | --- | --- | --- | --- | --- | --- | --- | --- |
|  | CA-CBT | UC | CA-CBT | UC | CA-CBT | UC |  |  |  |  |  |  |
| Outcome | (n=36) | (n=30) | (n=30) | (n=27) | (n=30) | (n=27) | CA-CBT | UC |  | Raw | FDR | d |
| Creatinine | 82.8 ± 14.7 | 81.7 ± 16.9 | 84.1 ± 14.9 | 84.9 ± 17.5 | 84.1 ± 13.3 | 81.9 ± 16.7 | 0.6 ± 1.4 | 1.6 ± 1.5 | -1.0 (-5.1 to 3.2) | .648 | .934 | 0.06 |
| Triglycerides | 1.1 ± 0.6 | 1.0 ± 0.5 | 1.1 ± 0.5 | 1.0 ± 0.5 | 1.1 ± 0.6 | 1.1 ± 0.7 | 0.0 ± 0.1 | 0.1 ± 0.1 | -0.1 (-0.3 to 0.1) | .382 | .934 | 0.15 |
| Cholesterol | 3.1 ± 0.6 | 3.2 ± 0.6 | 3.2 ± 0.7 | 3.3 ± 0.6 | 3.0 ± 0.6 | 3.3 ± 0.7 | -0.1 ± 0.1 | 0.1 ± 0.1 | -0.2 (-0.5 to 0.1) | .271 | .934 | 0.28 |
| HDL-cholesterol | 1.2 ± 0.3 | 1.3 ± 0.3 | 1.2 ± 0.3 | 1.4 ± 0.4 | 1.2 ± 0.3 | 1.4 ± 0.3 | 0.0 ± 0.0 | 0.1 ± 0.0 | -0.1 (-0.1 to 0.0) | .273 | .934 | 0.15 |
| LDL-cholesterol | 1.4 ± 0.4 | 1.5 ± 0.4 | 1.4 ± 0.5 | 1.5 ± 0.5 | 1.3 ± 0.5 | 1.5 ± 0.5 | -0.1 ± 0.1 | -0.0 ± 0.1 | -0.1 (-0.3 to 0.2) | .520 | .934 | 0.19 |
| hs-CRP | 0.8 ± 0.7 | 1.3 ± 1.9 | 0.7 ± 0.6 | 0.8 ± 1.1 | 0.7 ± 0.9 | 1.2 ± 2.6 | -0.1 ± 0.3 | -0.0 ± 0.3 | -0.1 (-0.9 to 0.8) | .868 | .934 | 0.05 |
| Non-HDL cholesterol | 1.9 ± 0.5 | 1.9 ± 0.5 | 1.9 ± 0.5 | 1.9 ± 0.6 | 1.8 ± 0.5 | 2.0 ± 0.8 | -0.1 ± 0.1 | 0.0 ± 0.1 | -0.1 (-0.4 to 0.2) | .429 | .934 | -0.20 |
| HbA1c | 40.3 ± 3.4 | 41.2 ± 9.3 | 40.0 ± 3.4 | 41.4 ± 10.4 | 39.7 ± 3.6 | 41.5 ± 9.5 | -0.4 ± 0.4 | -0.2 ± 0.5 | -0.2 (-1.4 to 1.1) | .799 | .934 | 0.02 |

**Note:** Raw scores are presented as mean±SD. Changes in least-square means from baseline to the three-month follow-up (primary endpoint) and the estimated difference at three months were derived from linear regression mixed models. The least-square mean changes are shown as estimated change ± SE. The estimated differences are presented with 95% confidence intervals. Effect sizes are shown as d, calculated as Cohen’s d. Units: Creatinine in mikromol/L; Triglycerides, Cholesterol HDL-cholesterol, LDL-cholesterol, Non-HDL cholesterol, HbA1c in mmol/L; hs-CRP in mg/L. **Abbrevations:** 3MFU: Three-month follow-up, CA-CBT: CBT for Cardiac Anxiety, UC: Usual Care, FDR: P-value corrected for the False Discovery Rate, HDL: **High-density** lipoprotein, LDL**: Low-density** lipoprotein, **CRP**: **C-reactive** protein, **HbA1c**: Haemoglobin A1c.

| **Supplemental Table 5.** Estimated within-group differences in mean change at post-treatment and at three-months follow-up. | | | | | | | | | | |
| --- | --- | --- | --- | --- | --- | --- | --- | --- | --- | --- |
| Raw scores | | | | | Estimated within-group change from baseline | | | | | |
| Outcome | Baseline  (n=48) | Post  (n=46) | 3MFU  (n=46) | 12MFU  (n=42) | Post | p | 3MFU | p | 12MFU | p |
| SAQ QoL | 45.8 ± 21.7 | 65.4 ± 20.3 | 65.9 ± 24.3 | 64.1 ± 21.9 | 19.0 (13.8 to 24.2) | <.001 | 19.9 (14.7 to 25.1) | <.001 | 18.1 (12.8 to 23.5) | <.001 |
| SAQ Physical^a^ | 82.8 ± 20.3 | 91.3 ± 15.7 | 90.8 ± 16.0 | 92.6 ± 14.9 | 9.0 (6.1 to 11.8) | <.001 | 7.4 (4.5 to 10.2) | <.001 | 8.6 (5.6 to 11.5) | <.001 |
| SAQ Stability | 81.2 ± 26.5 | 91.8 ± 21.8 | 89.1 ± 24.0 | 89.9 ± 22.8 | 10.5 (2.1 to 18.9) | .014 | 7.8 (-0.6 to 16.2) | .067 | 8.7 (0.1 to 17.3) | .049 |
| SAQ Frequency | 85.6 ± 16.9 | 89.8 ± 15.1 | 91.7 ± 14.2 | 91.7 ± 13.2 | 4.3 (-0.1 to 8.6) | .055 | 6.2 (1.9 to 10.5) | .005 | 6.1 (1.7 to 10.6) | .007 |
| CAQ | 30.1 ± 9.7 | 16.0 ± 9.1 | 17.3 ± 8.7 | 18.7 ± 9.7 | -13.8 (-16 to -11.7) | <.001 | -12.5 (-14.7 to -10.4) | <.001 | -11.1 (-13.3 to -8.9) | <.001 |
| PHQ-9 | 7.8 ± 6.0 | 4.8 ± 4.6 | 5.0 ± 5.3^b^ | 5.1 ± 5.4 | -2.9 (-4.1 to -1.6) | <.001 | -2.7 (-3.9 to -1.5) | <.001 | -2.4 (-3.7 to -1.2) | <.001 |
| GAD-7 | 6.1 ± 4.7 | 3.9 ± 3.8^b^ | 3.4 ± 3.3^b^ | 4.0 ± 4.7 | -2.4 (-3.4 to -1.3) | <.001 | -2.9 (-3.9 to -1.8) | <.001 | -2.2 (-3.3 to -1.2) | <.001 |
| BSQ | 36.5 ± 10.5 | 27.0 ± 6.0^b^ | 26.9 ± 7.7 ^b^ | 28.2 ± 8.2 | -9.6 (-12.1 to -7.2) | <.001 | -9.8 (-12.2 to -7.4) | <.001 | -8.7 (-11.2 to -6.3) | <.001 |
| GLTEQ | 71.4 ± 107.4 | 82.6 ± 131.5 ^b^ | 64.0 ± 91.3 ^b^ | 81.1 ± 119.7 | 10.5 (-31.3 to 52.4) | .620 | -8.0 (-49.9 to 33.8) | .704 | 8.4 (-34.6 to 51.5) | .699 |
| Health-care visits | 0.8 ± 1.1 | 0.5 ± 0.9 ^b^ | 0.5 ± 0.8 | 0.7 ± 1.3 | -0.3 (-0.7 to 0.1) | .136 | -0.3 (-0.7 to 0.1) | .108 | 0.0 (-0.4 to 0.4) | .898 |
| SCL frequency | 16.7 ± 8.4 | 13.0 ± 7.5^b^ | 12.7 ± 8.0 ^b^ | 11.3 ± 6.8 | -3.6 (-5.1 to -2.2) | <.001 | -3.9 (-5.4 to -2.5) | <.001 | -4.9 (-6.3 to -3.4) | <.001 |
| SCL severity | 14.8 ± 7.0 | 10.8 ± 6.4^b^ | 11.1 ± 7.1 ^b^ | 9.3 ± 5.5 | -4.0 (-5.2 to -2.7) | <.001 | -3.6 (-4.9 to -2.4) | <.001 | -5.0 (-6.3 to -3.7) | <.001 |
| SF-12 MCS | 43.1 ± 13.1 | 48.7 ± 11.3 ^b^ | 49.0 ± 11.0 ^b^ | 48.0 ± 12.0 | 5.5 (2.4 to 8.6) | .001 | 5.8 (2.7 to 8.9) | <.001 | 4.9 (1.7 to 8.0) | .003 |
| SF-12 PCS | 47.4 ± 8.5 | 50.3 ± 9.1^b^ | 49.4 ± 9.3 ^b^ | 50.1 ± 7.9 | 2.8 (0.9 to 4.8) | .005 | 1.8 (-0.1 to 3.8) | .066 | 1.8 (-0.2 to 3.8) | .075 |
| PSS-4 | 6.3 ± 3.0 | 4.6 ± 3.1^b^ | 4.9 ± 3.3^b^ | 5.7 ± 3.5 | -1.7 (-2.6 to -0.8) | <.001 | -1.5 (-2.4 to -0.5) | .002 | -0.7 (-1.7 to 0.2) | .128 |

**Note:** Raw scores are presented as mean±SD. The estimates are presented with 95% confidence intervals. **Abbreviations**: 3MFU: Three-month follow-up, 12MFU: Twelve-month follow-up, CA-CBT: CBT for Cardiac Anxiety, UC: Usual Care, SAQ: Seattle Angina Questionnaire, QoL: Quality of Life, CAQ: Cardiac Anxiety Questionnaire, BSQ: Body Sensation Questionnaire, PHQ-9: Patient Health Questionnaire, GAD-7: Generalized Anxiety Disorder 7-item scale. GLTEQ: The Godin Leisure-time Exercise Questionnaire. SCL: Symptom Checklist, SF-12 Physical Health Summary score, SF-12 PCS: Short-Form Health Survey, SF-12 MCS: Short-Form Health Survey Mental Health Summary score, PSS-4: Perceived Stress Scale-4. ^a^One outlying score removed at post-treatment assessment. ^b^ (n=45) One participant provided partial data due to perceived assessment burden. ^b^ (n=45) One participant provided partial data due to perceived assessment burden.

### **Supplemental Figure 1.** Waterfall Plots of change in SAQ subscales and Cardiac Anxiety Questionnaire from baseline to three-month follow-up

**A.**

**B.**

**C.**

**D.**

**E.**

**Legends**: Waterfall plots comparing individual-level changes from baseline to three-month follow-up (primary endpoint) between the CBT and UC-group on SAQ Quality of Life (panel A), SAQ Physical limitation (panel B), SAQ Angina frequency (panel C), SAQ Angina stability (panel D), and CAQ (Cardiac Anxiety; panel E) summary score. Each bar represents one participant and is ordered by magnitude of change; positive values indicate improvement and negative values indicate worsening, except for CAQ, where negative values indicate improvement. Bars are colored according to treatment group; CA-CBT (red) vs Usual Care (UC; green)

**Abbreviations:** CA-CBT: CBT for Cardiac Anxiety, UC: Usual Care, SAQ: Seattle Angina Questionnaire, QoL: Quality of Life, CAQ: Cardiac Anxiety Questionnaire

### **Supplemental Table 6.** Cohen’s d effect sizes for comparisons between CA-CBT and UC and CA-CBT within-group comparisons.

|  | Between-groups effect size  CA-CBT vs UC | | Within-group effect size  CA-CBT | |
| --- | --- | --- | --- | --- |
| **Outcome** | **d** | **95% CI** | **d** | **95% CI** |
| **SAQ QoL** |  |  |  |  |
| Post | 0.58 | (0.21 to 0.96) | 0.87 | (0.59 to 1.18) |
| 3MFU | 0.53 | (0.10 to 0.96) | 0.91 | (0.57 to 1.26) |
| 12MFU |  |  | 0.83 | (0.53 to 1.15) |
| **SAQ physical ^a^** |  |  |  |  |
| Post | 0.45 | (0.20 to 0.71) | 0.44 | (0.24 to 0.65) |
| 3MFU | 0.28 | (0.04 to 0.51) | 0.36 | (0.19 to 0.54) |
| 12MFU |  |  | 0.42 | (0.20 to 0.64) |
| **SAQ stability** |  |  |  |  |
| Post | 0.46 | (-0.06 to 0.98) | 0.40 | (-0.02 to 0.82) |
| 3MFU | 0.18 | (-0.30 to 0.66) | 0.29 | (-0.08 to 0.67) |
| 12MFU |  |  | 0.33 | (-0.10 to 0.75) |
| **SAQ frequency** |  |  |  |  |
| Post | 0.29 | (-0.09 to 0.68) | 0.25 | (-0.04 to 0.54) |
| 3MFU | 0.19 | (-0.23 to 0.61) | 0.37 | (0.04 to 0.70) |
| 12MFU |  |  | 0.36 | (-0.01 to 0.74) |
| **CAQ** |  |  |  |  |
| Post | 1.01 | (0.56 to 1.46) | 1.43 | (1.03 to 1.82) |
| 3MFU | 0.84 | (0.39 to 1.30) | 1.29 | (0.91 to 1.68) |
| 12MFU |  |  | 1.15 | (0.75 to 1.54) |
| **PHQ-9** |  |  |  |  |
| Post | 0.36 | (0.04 to 0.69) | 0.47 | (0.24 to 0.70) |
| 3MFU ^b^ | 0.32 | (-0.02 to 0.66) | 0.45 | (0.17 to 0.73) |
| 12MFU |  |  | 0.40 | (0.13 to 0.67) |
| **GAD-7** |  |  |  |  |
| Post ^b^ | 0.17 | (-0.20 to 0.55) | 0.51 | (0.21 to 0.80) |
| 3MFU ^b^ | 0.33 | (-0.06 to 0.72) | 0.61 | (0.29 to 0.94) |
| 12MFU |  |  | 0.48 | (0.19 to 0.77) |
| **BSQ** |  |  |  |  |
| Post ^b^ | 0.64 | (0.20 to 1.07) | 0.92 | (0.55 to 1.30) |
| 3MFU ^b^ | 0.45 | (0.02 to 0.89) | 0.94 | (0.58 to 1.29) |
| 12MFU |  |  | 0.84 | (0.44 to 1.23) |
| **Health-care visits** |  |  |  |  |
| Post ^b^ | 0.24 | (-0.17 to 0.64) | 0.27 | (-0.11 to 0.64) |
| 3MFU ^b^ | 0.14 | (-0.33 to 0.60) | 0.29 | (-0.15 to 0.73) |
| 12MFU |  |  | 0.02 | (-0.41 to 0.46) |
| **SCL frequency** |  |  |  |  |
| Post ^b^ | 0.11 | (-0.16 to 0.39) | 0.44 | (0.24 to 0.63) |
| 3MFU ^b^ | 0.05 | (-0.23 to 0.33) | 0.47 | (0.25 to 0.69) |
| 12MFU |  |  | 0.58 | (0.33 to 0.83) |
| **SCL severity** |  |  |  |  |
| Post ^b^ | 0.12 | (-0.19 to 0.42) | 0.57 | (0.36 to 0.77) |
| 3MFU ^b^ | -0.03 | (-0.34 to 0.28) | 0.52 | (0.29 to 0.75) |
| 12MFU |  |  | 0.72 | (0.45 to 0.99) |
| **SF-12 MCS** |  |  |  |  |
| Post ^b^ | 0.21 | (-0.15 to 0.58) | 0.42 | (0.18 to 0.67) |
| 3MFU ^b^ | 0.27 | (-0.09 to 0.63) | 0.44 | (0.17 to 0.72) |
| 12MFU |  |  | 0.37 | (0.06 to 0.68) |
| **SF-12 PCS** |  |  |  |  |
| Post ^b^ | 0.29 | (-0.04 to 0.63) | 0.33 | (0.10 to 0.56) |
| 3MFU ^b^ | 0.06 | (-0.27 to 0.40) | 0.22 | (-0.01 to 0.44) |
| 12MFU |  |  | 0.21 | (-0.07 to 0.49) |
| **GLTEQ** |  |  |  |  |
| Post ^b^ | 0.09 | (-0.45 to 0.63) | 0.10 | (-0.29 to 0.48) |
| 3MFU ^b^ | -0.33 | (-0.90 to 0.23) | -0.07 | (-0.44 to 0.29) |
| 12MFU |  |  | 0.08 | (-0.36 to 0.51) |
| **PSS4** |  |  |  |  |
| Post ^b^ | 0.19 | (-0.22 to 0.61) | 0.57 | (0.24 to 0.90) |
| 3MFU ^b^ | 0.22 | (-0.17 to 0.61) | 0.48 | (0.18 to 0.79) |
| 12MFU |  |  | 0.24 | (-0.09 to 0.57) |

**Note:** Effect sizes are shown as d, calculated by Cohen’s d. **Abbreviations**: 3MFU: Three-month follow-up, CA-CBT: CBT for Cardiac Anxiety, UC: Usual Care, SAQ: Seattle Angina Questionnaire, QoL: Quality of Life, CAQ: Cardiac Anxiety Questionnaire, BSQ: Body Sensation Questionnaire, PHQ-9: Patient Health Questionnaire, GAD-7: Generalized Anxiety Disorder 7-item scale. GLTEQ: The Godin Leisure-time Exercise Questionnaire, SCL: Symptom Checklist, SF-12 Physical Health Summary score, SF-12 PCS: Short-Form Health Survey, SF-12 MCS: Short-Form Health Survey Mental Health Summary score, PSS-4: Perceived Stress Scale-4. ^a^One outlying score removed at post-treatment assessment. ^b^ (n=45) One participant provided partial data due to perceived assessment burden.

## Cardiac specific health-care utilization

At the three‑month follow‑up, there were no significant between‑group differences in cardiac‑specific health‑care utilization measured with the AFSS. The estimated difference in mean change from baseline to follow‑up was 0.7 visits (95% CI: 0.3 to 1.5; FDR‑adjusted p = .471; Table 2).

## Usual care context – health-care visits

At post‑treatment, health‑care visits during the preceding four weeks was comparable across groups. General practitioner visits were reported by 14 participants (29%) in the UC group and 7 participants (15%) in the CA‑CBT group. Cardiologist visits were reported by 7 participants (15%) in UC and 12 participants (25%) in CA‑CBT. Visits to other physicians occurred in 6 participants (13%) in UC and 8 participants (17%) in CA‑CBT. Nurse contacts were more frequent in the UC group, reported by 19 participants (40%) compared with 11 participants (23%) in CA‑CBT. Physiotherapy visits were reported by 7 participants (15%) in each group. Visits with a social worker were uncommon, reported by 3 participants (6%) in UC and 1 participant (2%) in CA‑CBT.

At the 3‑month follow‑up, patterns of health‑care utilization were similar. General practitioner visits occurred in 8 participants (17%) in UC and 10 participants (21%) in CA‑CBT. Cardiologist visits were reported by 6 participants (13%) in the UC group and 8 participants (17%) in the CA‑CBT group. Contacts with other physicians were reported by 6 participants (13%) in UC and 3 participants (6%) in CA‑CBT. Nurse visits were reported by 12 participants (25%) in the UC group and 10 participants (21%) in the CA‑CBT group. Physiotherapy contacts occurred in 7 participants (15%) in UC and 5 participants (10%) in CA‑CBT. Social work and other provider contacts remained rare.

## Psychologist/psychotherapist visits

Four (8%) participants in each arm reported having attended occasional visits with a psychologist/psychotherapist for psychosocial support at post‑treatment. Psychologist/psychotherapist visits at three-months were 2 (4%) in the CA‑CBT group and 3 (6%) in the UC group. Contacts with psychologist/psychotherapist were brief supportive contacts addressing general psychosocial stressors, e.g. family members illness or marriage counseling. These supportive contacts were not focused on cardiac anxiety and *not* considered structured psychological treatment; therefor not classified as concurrent treatment.

## Change in psychotropic medication

Information on concurrent psychological treatment and changes in psychotropic medication was assessed at both post‑treatment and 3‑month follow‑up. No changes in SSRI or other psychotropic medications occurred between baseline and post‑treatment in either group. One participant in the UC group reported an increased dosage of benzodiazepine at the three‑month follow‑up.

## *Changes in cardiac health*

Three participants in the CA-CBT group self-reported a deterioration in cardiac health at post-treatment. One participant was hospitalized for cardiac care, while two reported diffuse symptoms, including chest tightness and a general decline in physical condition. At the three-month follow-up, five participants in the CA-CBT group reported worsening cardiac health: two reported diffuse symptom changes, one had ECG changes, one underwent PCI, and one experienced a suspected muscle spasm. At post-treatment, six participants in the UC group reported a deterioration in cardiac health. Five of these cases involved diffuse symptoms, such as lack of energy, heart racing, and stinging sensations, while one participant reported extra heartbeats. At the three-month follow-up, seven participants in the UC group reported worsening cardiac health: five experienced an increase in diffuse symptoms, one reported angina, and one had increased atrial fibrillation.

# References

1 Eifert, G. H., Zvolensky, M. J. & Lejuez, C. W. Heart-focused anxiety and chest pain: A conceptual and clinical review. *Clinical Psychology: Science and Practice* **7**, 403–417 (2000). <https://doi.org/10.1093/clipsy.7.4.403>

2 Association, A. P. (Text Revision. Washington, DC: American Psychiatric Association, 2022).

3 Edmondson, D. An Enduring Somatic Threat Model of Posttraumatic Stress Disorder Due to Acute Life-Threatening Medical Events. *Soc Personal Psychol Compass* **8**, 118–134 (2014). <https://doi.org/10.1111/spc3.12089>

4 Princip, M., Ledermann, K. & von Känel, R. Posttraumatic Stress Disorder as a Consequence of Acute Cardiovascular Disease. *Curr Cardiol Rep* **25**, 455–465 (2023). <https://doi.org/10.1007/s11886-023-01870-1>

5 Eifert, G. H. *et al.* The Cardiac Anxiety Questionnaire: development and preliminary validity. *Behaviour Research and Therapy* **38**, 1039–1053 (2000). <https://doi.org/https://doi.org/10.1016/S0005-7967(99)00132-1>

6 Leissner, P., Held, C., Rondung, E. & Olsson, E. M. G. The factor structure of the cardiac anxiety questionnaire, and validation in a post-MI population. *BMC Medical Research Methodology* **22**, 338 (2022). <https://doi.org/10.1186/s12874-022-01820-5>

7 Cohen, S., Kamarck, T. & Mermelstein, R. A global measure of perceived stress. *Journal of health and social behavior*, 385–396 (1983).

8 Jenkins, L. Test specifications for the Bubien and Kay (revised Jenkins) symptom checklist: frequency and severity. *Baltimore, MD: University of Maryland* (1993).

9 Dorian, P. *et al.* Validation of a new simple scale to measure symptoms in atrial fibrillation: the Canadian Cardiovascular Society Severity in Atrial Fibrillation scale. *Circulation: Arrhythmia and Electrophysiology* **2**, 218–224 (2009).

10 Chambless, D. L., Caputo, G. C., Bright, P. & Gallagher, R. Assessment of fear of fear in agoraphobics: the body sensations questionnaire and the agoraphobic cognitions questionnaire. *J Consult Clin Psychol* **52**, 1090–1097 (1984). <https://doi.org/10.1037//0022-006x.52.6.1090>

11 Amireault, S. & Godin, G. The Godin-Shephard leisure-time physical activity questionnaire: validity evidence supporting its use for classifying healthy adults into active and insufficiently active categories. *Perceptual and motor skills* **120**, 604–622 (2015).

12 Kroenke, K., Spitzer, R. L. & Williams, J. B. W. The PHQ-9. *Journal of General Internal Medicine* **16**, 606–613 (2001). <https://doi.org/https://doi.org/10.1046/j.1525-1497.2001.016009606.x>

13 Spitzer, R. L., Kroenke, K., Williams, J. B. W. & Löwe, B. A Brief Measure for Assessing Generalized Anxiety Disorder: The GAD-7. *Archives of Internal Medicine* **166**, 1092–1097 (2006). <https://doi.org/10.1001/archinte.166.10.1092>

14 Failde, I., Medina, P., Ramírez, C. & Arana, R. Assessing health-related quality of life among coronary patients: SF-36 vs SF-12. *Public Health* **123**, 615–617 (2009). <https://doi.org/10.1016/j.puhe.2009.07.013>

15 Hakkaart-van Roijen, L., Van Straten, A., Donker, M. & Tiemens, B. Manual Trimbos/iMTA questionnaire for Costs associated with Psychiatric illness (TiC-P). *Institute for Medical Technology Assessment* (2002).

16 Migueles, J. H. *et al.* Accelerometer Data Collection and Processing Criteria to Assess Physical Activity and Other Outcomes: A Systematic Review and Practical Considerations. *Sports Med* **47**, 1821–1845 (2017). <https://doi.org/10.1007/s40279-017-0716-0>

17 Sandborg, J. *et al.* Effectiveness of a Smartphone App to Promote Healthy Weight Gain, Diet, and Physical Activity During Pregnancy (HealthyMoms): Randomized Controlled Trial. *JMIR Mhealth Uhealth* **9**, e26091 (2021). <https://doi.org/10.2196/26091>

18 Hildebrand, M., VT, V. A. N. H., Hansen, B. H. & Ekelund, U. Age group comparability of raw accelerometer output from wrist- and hip-worn monitors. *Med Sci Sports Exerc* **46**, 1816–1824 (2014). <https://doi.org/10.1249/mss.0000000000000289>
